# One-dimensional anionic radical chains based on M_2_P_2_ rings (M = Ti, Zr, Hf)†

**DOI:** 10.1039/d6sc04762a

**Published:** 2026-07-30

**Authors:** Xiaofei Sun, Ravi Yadav, Alexander Hinz, Jörg Göttlicher, Tonya Vitova, Sabrina Dinauer, Manfred Scheer, Peter W. Roesky

**Affiliations:** a Institut für Anorganische Chemie (AOC), Karlsruhe Institute of Technology (KIT) Kaiserstr. 12 Karlsruhe 76131 Germany roesky@kit.edu; b Department of Chemistry, Indian Institute of Technology Roorkee Roorkee Uttarakhand 247667 India; c Institut für Photonenforschung und Synchrotronstrahlung (IPS), Karlsruhe Institute of Technology (KIT) Kaiserstr. 12 Karlsruhe 76131 Germany; d Institute for Nuclear Waste Disposal (INE), Karlsruhe Institute of Technology (KIT) Kaiserstr. 12 Karlsruhe 76131 Germany; e Institut für Anorganische Chemie, Universität Regensburg Regensburg 93040 Germany; f Institute for Nanotechnology, Karlsruhe Institute of Technology (KIT) Kaiserstr. 12 Karlsruhe 76131 Germany

## Abstract

This work deals with the synthesis of metallocene-phosphorus based radical coordination polymers. The radical polymeric chains were synthesized by a single step reaction between commercially available group 4 metallocene dichlorides [Cp_2_MCl_2_] (M = Ti, Zr, Hf; Cp = η^5^-C_5_H_5_), pentaphosphaferrocene [Cp*Fe(η^5^-P_5_)] (Cp* = η^5^-C_5_Me_5_) and potassium metal. These reactions selectively gave polymeric chains of radical, [K{Cp_2_M}_2_{(η^4^-P_5_)FeCp*}_2_]_*n*_ (M = Ti, Zr, Hf). The four membered P_2_M_2_ core is the first example of such a phosphorus-based ring with a radical anionic nature. Moreover, it is also a rare example of a main group element based radical polymeric chain. The radical nature of the metallopolymer is further established by EPR, XANES and quantum chemical calculations. The potassium ion acts as a linker ion between two [{Cp_2_M}{(η^4^-P_5_)FeCp*}] fragments. The reaction of [K{Cp_2_M}_2_{(η^4^-P_5_)FeCp*}_2_]_*n*_ with [2.2.2]-cryptand in THF resulted in chain cleavage into charge separated monomeric complexes [(K[2.2.2]-cryptand){Cp_2_M}_2_{(η^4^-P_5_)FeCp*}_2_] (M = Ti, Zr, Hf).

## Abstract 2

This work deals with the synthesis of metallocene-phosphorus based radical coordination polymers.

## Graphical abstract

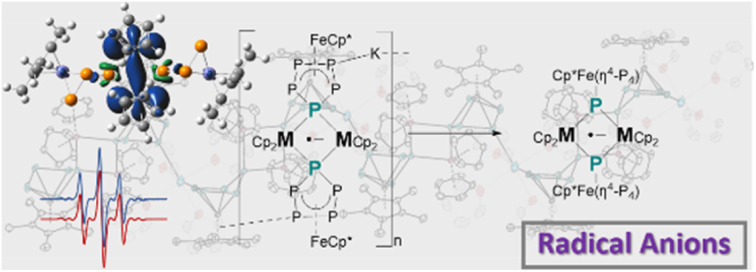

## Introduction

Radicals are open-shell compounds having unpaired electrons. They play crucial roles as intermediates in chemical and biological processes and also find applications in diverse areas, including organic synthesis and materials science.^1–3^ Since the report on the first organic radical Ph_3_C˙ in 1900 by Gomberg,^4^ numerous radicals have been isolated. Compared to C-, N- or O-based first-row p-block radicals, radical species with heavy p-block elements tend to be more transient and are less known.^5–12^ This is due to their high reactivity, which often leads to dimerization or bond activation. Consequently, the synthesis and isolation of stable heavy p-block or inorganic radicals remains challenging and requires kinetic and electronic stabilization methods. Stable phosphorus-centered radicals represent a significantly interesting class of compounds in contemporary inorganic chemistry owing not only to their novel structure motifs but also to their unusual reactivities and physical properties. Over the past few decades, neutral, cationic, and anionic phosphorus-centered radicals have been structurally characterized. These compounds were comprehensively reviewed by Kundu^6^ and Budnikova *et al.*^9^ Among these, four-membered cyclic radicals composed of phosphorus and other p-block elements which display diphosphacyclobutane-type scaffolds are interesting compounds due to their potential application in various fields such as catalytic transformations including small molecule activation and conversion, as well as thermal and photochemical coupling reactions.^3,13,14^

**Fig. 1:**
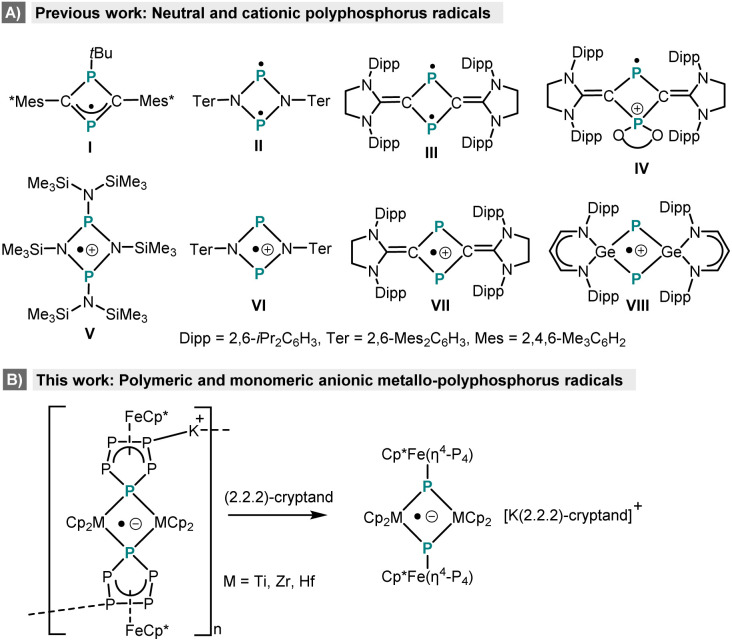
A) Selected examples of phosphorus-based P_2_E_2_-four-membered cyclic neutral and cationic radicals, and B) the radical anions from this work.

In a pioneering study of Niecke *et al.* in 1995, air-stable 1,3-diphosphacyclobutane-2,4-diyl was reported, featuring carbon-centered singlet biradical character.^15^ In 2006, Ito *et al.* prepared an air-tolerant 1,3-diphosphacyclobuten-4-yl neutral radical with a planar P_2_C_2_ scaffold (I, Fig. 1).^16^ The very first example of a cyclic radical cation which is a cyclodiphosphazene was disclosed by Wang *et al.* in 2014 (V, Fig. 1).^17^ Also, P_2_E_2_ (E = B, C, N, Al, Ge) type neutral and cationic radicals were reported by Bertrand,^18^ Schnöckel,^19–21^ Ghadwal,^22^ Bresien,^23^ Schulz,^23–26^ Li,^27,28^ Grützmacher,^28^ Su^28^ and Wang *et al.* (*cf.* selected examples, II–IIIV, Fig. 1)^17,29^ In contrast, polymeric stable heavy p-block radicals with a cyclobutane-type scaffold are extremely rare. In 2019, Tan and Wang *et al.* reported the first example of a heavy main group element-based boron radical polymer with B_2_P_2_ chain links.^30^ To the best of our knowledge, neither related four-membered cyclic phosphorus-based radical anions nor the corresponding dimetalla-radical anions (P_2_M_2_) have been reported to date.

[Cp*Fe(η^5^-P_5_)]^31^ (Cp* = η^5^-C_5_Me_5_) is an air-stable sandwich-type pentaphosphaferrocene, which has been frequently used as a phosphorus source to construct novel compounds, ranging from molecular species to coordination polymers.^32–39^ The *cyclo*-P_5_ ring of [Cp*Fe(η^5^-P_5_)] participates in redox events.^40–46^ We were interested in developing a synthetic pathway for accessing a phosphorus-based radical polymer. For that purpose, we envisaged that [Cp*Fe(η^5^-P_5_)] could be an ideal starting material owing to its redox properties and its possibility as an efficient linker in metallopolymers. By taking advantage of the redox non-innocent nature of the *cyclo*-P_5_ ring and structural flexibility of the five phosphorus nuclei, we designed the synthesis to form a series of unprecedented phosphorus/transition metal radical polymers assembled by K cations (Fig. 1B, left). These room-temperature stable 1,3-diphospha-2,4-dimetalla-radical anions represent the first ever example of a phosphorus/transition metal-based radical polymer and the first example of a cyclic P_2_E_2_ radical anion.

## Results and discussion

### Synthesis

**Scheme 1:**
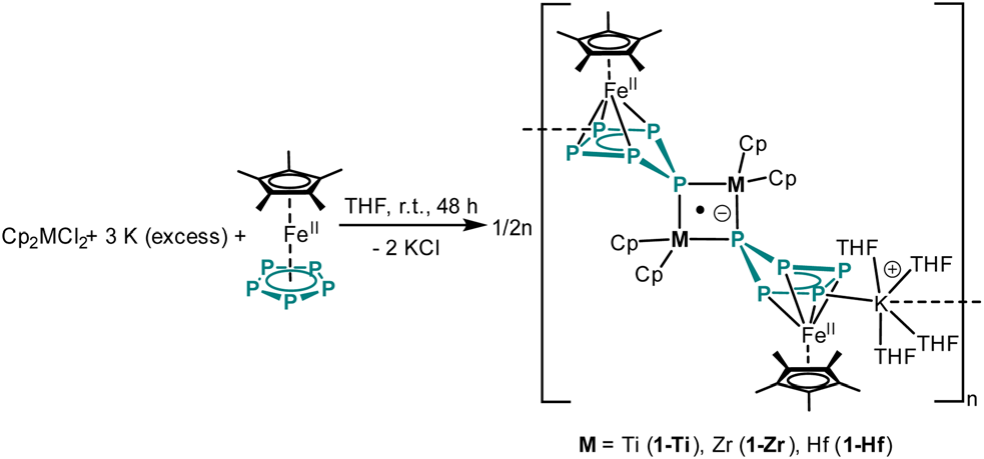
Synthesis of the mixed-valent group 4 radical coordination polymers 1-M.

In this study, we report a one-pot reaction for the synthesis of a series of novel mixed-valent group 4 radical coordination polymers. The commercially available group 4 metallocene dichlorides [Cp_2_MCl_2_] (M = Ti, Zr, Hf; Cp = η^5^-C_5_H_5_), pentaphosphaferrocene [Cp*Fe(η^5^-P_5_)]^31^ and elemental potassium in THF were reacted at ambient temperature (Scheme 1). The reaction mixtures turned from green (the color due to [Cp*Fe(η^5^-P_5_)]) to dark purple (for Zr) or dark brown (for Ti and Hf). After stirring overnight, the resulting solutions were filtered and concentrated. Single crystals could be obtained by slow diffusion of *n*-pentane into their respective THF solutions with yields of 57% (1-Ti), 49% (1-Zr) and 47% (1-Hf). The radical coordination polymers were isolated as dark yellow (Ti), dark purple (Zr) and brown (Hf) crystals, respectively. Interestingly, the molecular DME containing compound [K(dme)_4_][{Cp*Fe(η^4^-P_5_)MCp_2_}_2_] (1-Zr′) was obtained as red crystals by using preformed [Cp*Fe(η^5^-P_5_)]^2−^ ^46^ in DME by reacting [Cp*Fe(η^5^-P_5_)] (1 eq) with an excess amount of KC_8_ (2.5 eq) and adding [Cp_2_ZrCl_2_] at −50 °C (67% yield) or room temperature (37% yield) to the reaction solution (*cf.* the SI). However, in this way, it was not possible to obtain the related molecular complexes of Ti and Hf.

**Fig. 2:**
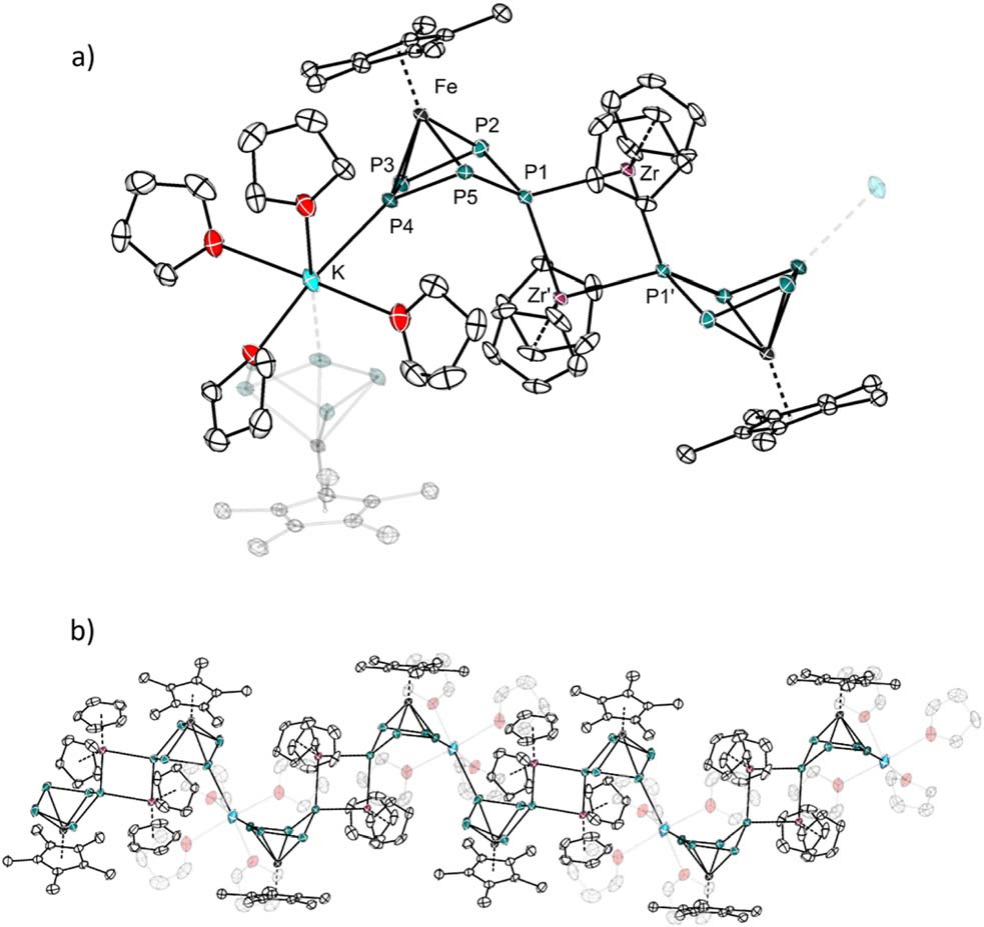
a) The structure of the repeating unit in the radical coordination polymer 1-Zr. Hydrogen atoms and the non-coordinating THF molecule are omitted for clarity. b) Depiction of the polymeric structure of 1-Zr. Depicted with thermal ellipsoids of 40%. Selected bond lengths and angles are summarized in Table S2 and in the SI.

Single crystal X-ray diffraction analyses reveal that the three polymeric radicals (1-M) are isostructural, crystallizing in the monoclinic space group *C*2/*c* and exhibiting a one-dimensional zig-zag chain structure (Fig. 2). The polymeric chain structure is built by self-assembly of the monomeric units by linkage of the potassium cation between the polyphosphide entities. The asymmetric units contain one Cp_2_M, one [(η^4^-P_5_)FeCp*] and half of a potassium cation, which is coordinated by two THF molecules. The central structure motifs consist of almost ideal rectangular planar {M_2_P_2_} four-membered rings. The respective angles and M–P bond lengths are summarized in Table S2 (see the SI). Upon reduction, the *cyclo-*P_5_ ring adopts an envelope geometry, with four P atoms (P2–P5) being in a plane coordinating to the {Cp*Fe} fragment, while P1 is being out of the P_4_ plane and coordinated to the two metal atoms. The P–P bond lengths of 2.1395(9)–2.2054(8) Å are within the range of typical single and double bonds.^47^ One prominent structure feature is relatively long P1–P2 (2.1898–2.1993 Å) and P1–P5 (2.1961–2.2054 Å) bonds, when comparing it to other compounds with bent *cyclo*-P_5_ rings.^48,49^

**Scheme 2:**
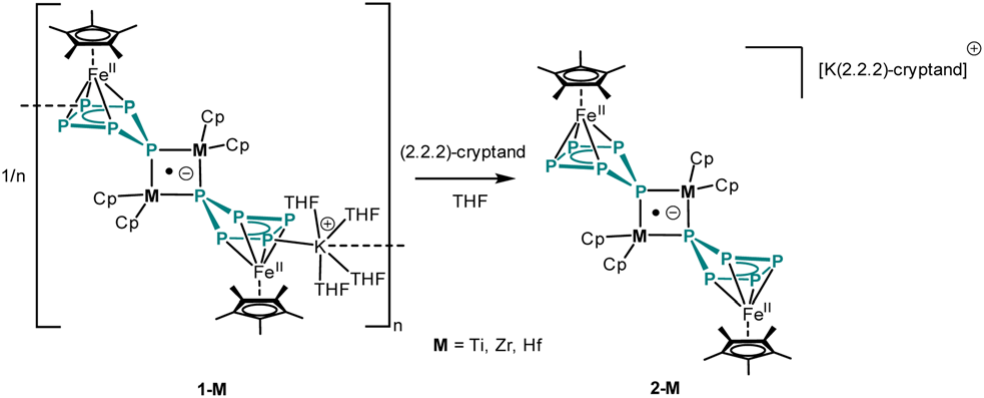
Conversion of the polymeric compounds 1-M to the monomeric analogs 2-M.

**Fig. 3:**
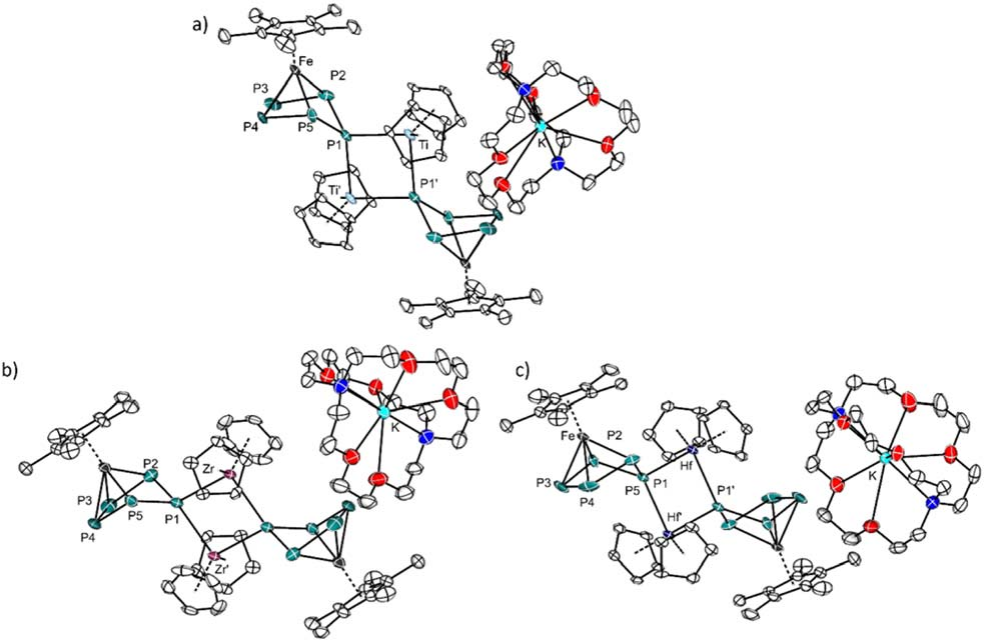
Molecular structures of 2-Ti (a), 2-Zr (b) and 2-Hf (c). Depicted with thermal ellipsoids of 40%. Selected bond lengths and angles are summarized in Table S2 in the SI.

Compared to the polymeric structures of 1-M, complex 1-Zr′ is best described as a charge-separated species. The potassium cation is coordinated by four DME molecules and the anionic part [{Cp*Fe(η^4^-P_5_)ZrCp_2_}_2_]^−^ is similar to that in 1-Zr, with comparable Zr–P bond distances. However, the coordination geometry of the Zr_2_P_2_ core in 1-Zr′ shows a slightly greater deviation from the ideal octahedral angle (see SI, Table S2). The polymeric compounds 1-M were converted into the respective monomeric species [K(2.2.2)-crypt][{Cp*Fe(η^4^-P_5_)MCp_2_}_2_] (2-M) by treatment with [2.2.2]-cryptand in THF in moderate yields (Scheme 2). The monomeric species were characterized by single crystal X-ray diffraction analysis and all are isostructural, crystallizing in the monoclinic space group *C*2/*c*. The molecular structures are depicted in Fig. 3 and some important structural parameters are listed in Table S2 (see the SI). Compounds 2-M crystallize as charge-separated ion pairs. The potassium cation is coordinated by the chelating cryptand and no interaction can be found with the radical anion. Within the radical anions, there are negligible changes in the structural parameters as compared with their polymeric analogs 1-M. All complexes 1-M and 2-M are virtually insoluble in solvents such as *n*-pentane, *n*-hexane and toluene but readily dissolve in THF. Notably, although compounds 1-M were obtained as coordination polymers in the solid state, in solution, their behavior is likely different. Since all complexes readily dissolve in THF, it is reasonable that the polymeric chains dissociate into smaller units under this condition.

Dissolving the crystals of 1-M followed by recrystallization again afforded the coordination polymers, indicating that this process is reversible. Unfortunately, diffusion-ordered NMR spectroscopy (DOSY) experiments did not lead to meaningful results due to the paramagnetic nature of all compounds. Nevertheless, the very similar NMR and EPR spectra of 1-Zr and 2-Zr (see SI, Fig. S18, S21, S24, and S26) suggest that they resemble closely related motifs in solution. The use of [2.2.2]-cryptand primarily serves to sequester the potassium cation, thereby enabling the crystallization of the monomeric species 2-M.

### Spectroscopic and theoretical calculations

**Fig. 4:**
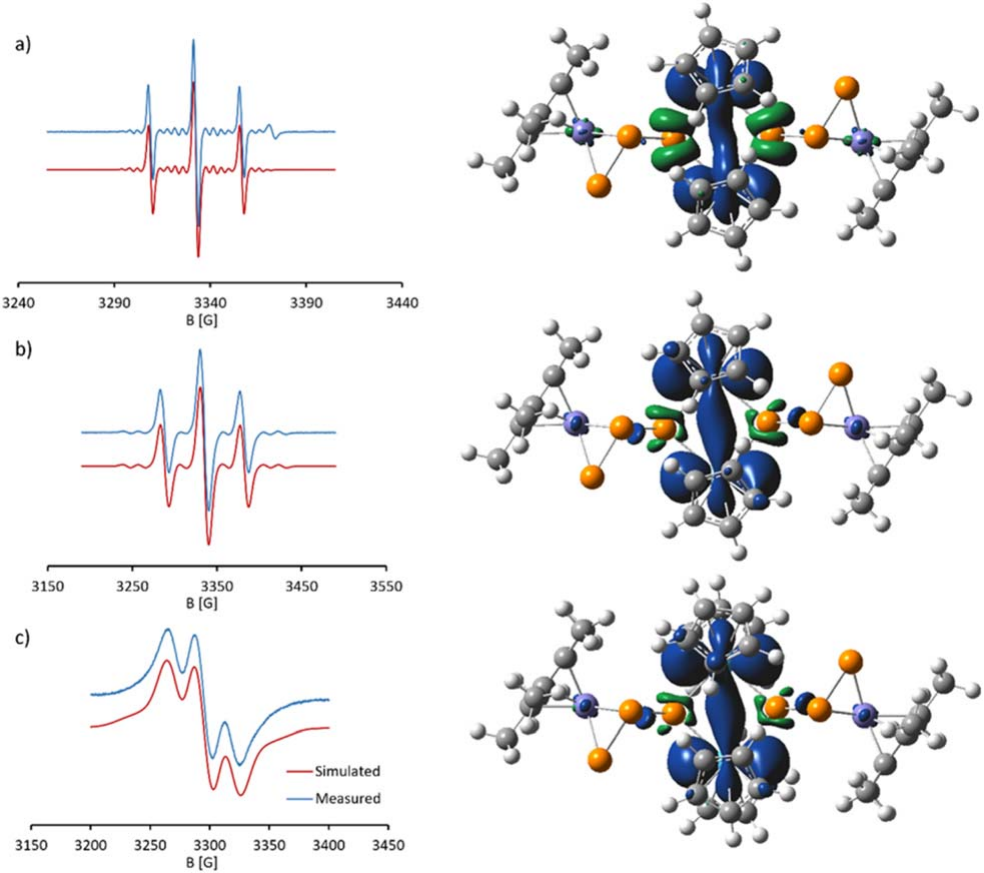
EPR spectra of 1-Ti (a), 1-Zr (b) and 1-Hf (c) in THF solution at ambient temperature (measured top and simulated bottom) and the spin density distribution of the respective radical anions (isovalue of 0.0012).^51–54^

To get deeper insight into the electronic structure of the radical species, the electron paramagnetic resonance (EPR) spectra of all seven compounds 1-M, 1-M′and 2-M were recorded in THF at ambient temperature (Fig. 4 and see SI, S17–S23). For all three radicals, the EPR spectra display a triplet signal due to hyperfine coupling with two equivalent phosphorus nuclei (*I* = ½, 100%) at *g* = 2.047 (1-Ti), 2.014 (1-Zr) and 2.039 (1-Hf), 2.002 (2-Ti), 2.013 (2-Zr), 2.042 (2-Hf) and 2.009 (1-Zr′), respectively. Satellite signals arising from the interaction of the unpaired electron with spin active ^47^Ti (*I* = 5/2, 7.4%), ^49^Ti (*I* = 7/2, 5.4%) and ^91^Zr isotopes (*I* = 5/2, 11.2%) are also observed for 1-Ti, 1-Zr, 2-Ti, 2-Zr and 1-Zr′. Due to the larger line broadening for 1-Hf and 2-Hf, no satellite signals could be resolved, but the corresponding hyperfine coupling parameter was nevertheless included in the simulation. The spectra were reasonably well simulated and the values of the phosphorus hyperfine coupling constants of *A*_iso_ = 68.1 (1-Ti), 67.2 (2-Ti), 66.4 (1-Zr), 66.4 (2-Zr), 66.3 (1-Zr′), 62.3 MHz (1-Hf) and 62.2 MHz (2-Hf) decrease from Ti to Hf while the values of the transition metal hyperfine coupling constants of *A*_iso_ = 11.4 (1-Ti), 11.0 (2-Ti), 25.3 (1-Zr), 25.7 (2-Zr), 25.4 (1-Zr′), 33.2 MHz (1-Hf) and 32.7 MHz (2-Hf) increase from Ti to Hf. When comparing the same metal, the EPR spectra of 1-M (1-M′) and 2-M are similar, indicating a closely related electronic structure of the radical species. Magnetic susceptibility was additionally determined using the Evans method in solution.^50^ The magnetic moments of 1-Ti, 1-Zr, 1-Zr′ and 1-Hf were found to be 1.75, 1.64, 1.80, and 1.78 *µ*_B_, corresponding to 1.02, 0.92, 1.06 and 1.04 unpaired electrons, respectively. These values are consistent with each other and in good agreement with the EPR spectroscopic data, supporting the presence of one unpaired electron across the series. ^1^H NMR spectra were recorded for 1-Zr, 1-Zr′ and 2-Zr in THF-*d*_8_. Except for the signals for the coordinated THF (1-Zr), DME (1-Zr′) and cryptand (2-Zr), only the proton signals of the Cp* ligand were detected as broad signals at 0.42, 0.41 and 0.40, respectively. For the Cp ligands, no signal was detected. The ^31^P{^1^H} NMR spectra remained silent. It is apparent that the spin density is delocalized within the M_2_P_2_ four-membered ring, which indicates some degree of M(III) character in the radical anion. The computed SOMOs (Fig. S27) reflect this, as the dominant contributions to the orbital comprise d_z^2^_ character of the Ti, Zr or Hf atoms, respectively (Gaussian16, PBE0/Def2-SVP/D3BJ).^51–54^ The resulting overall spin density features major components on these metal atoms approximating 0.5*e* each (0.55–0.42*e*), while minor densities are found on the bridging P atoms (0.06–0.02*e*) (Fig. 4). Despite the appearance of the spin density distribution, there is no transannular bond. QTAIM analysis shows a ring critical point at the centre of the M_2_P_2_ moiety and therefore a minimum of electron density, consistent with no direct bonding interaction (see Fig. S29).^55^ Additionally, AIM charges were calculated for the mixed valent [{Cp*Fe(η^4^-P_5_)MCp_2_}_2_]^−^ monoanions. The charge distribution shows equal delocalization of the electron on both metals, thus corroborating a Robin-Day class III mixed-valence complex (see Fig. S29).

### UV/Vis and XANES spectroscopy

**Fig. 5:**
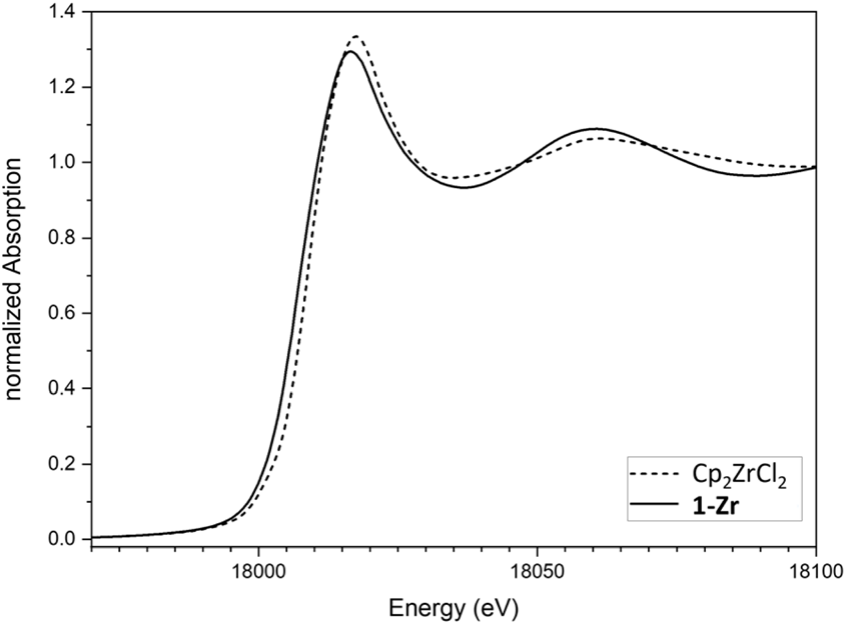
Transmission Zr K-edge XANES spectra of compound 1-Zr (solid line) and of a Zr(iv) reference compound Cp_2_ZrCl_2_ (dashed line).

The UV/Vis spectra of the radical anions 1-M were recorded in THF (see SI, Fig. S14–S16). The respective complexes show two broad absorptions in the visible region. The Zr complex 1-Zr exhibits a strong absorption band at 528 nm and a weaker one at 670 nm, consistent with the purple appearance of 1-Zr in solution. In comparison, the Ti and Hf complexes 1-Ti and 1-Hf show an intense absorption at 465 nm (1-Ti) and 495 nm (1-Hf) and a weaker one at 628 nm (1-Ti) and 648 nm (1-Hf). The blue-shifted absorptions of 1-Ti and 1-Hf are consistent with their olive greenish appearance in solution. Zr K-edge X-ray absorption near edge structure (XANES) spectra were recorded to gain information about the Zr formal oxidation state in the coordination polymer 1-Zr. In comparison, [Cp_2_ZrCl_2_] was chosen as a Zr(iv) reference compound (Fig. 5). The shift of the absorption edge of the spectrum of 1-Zr is about 1.6 eV ± 0.25 eV towards lower energy, which indicates contribution of the lower Zr oxidation state. It is also possible that the electron density on the Zr atom in 1-Zr is higher than that on the Zr atom in the reference [Cp_2_ZrCl_2_] compound.^56^ The energy shift was measured for the normalized spectra at the rising absorption edge at 0.5 absorption located at 18 005.5 eV and 18 007.1 eV for the spectra of 1-Zr and [Cp_2_ZrCl_2_], respectively. This Z K-edge XANES result is consistent with the EPR results.

It is noteworthy that the structural parameters of 1-M and 2-M agree well with those of [{Cp*Fe(η^4^-P_5_)] doubly reduced fragments^46^ (averaged Fe–P bond lengths in each case: 2.30 Å), which leaves merely one additional electron delocalized in the M_2_P_2_ scaffold that evokes only small changes in the M oxidation state.

## Conclusions

In this work, we reported the synthesis and characterization of unprecedented one-dimensional radical coordination polymers based on group 4 metallocenes (Ti, Zr, and Hf) and polyphosphorus ligands. These radical polymers were synthesized from a one pot reaction and feature a mixed-valent group-4-metal-based M_2_P_2_ central core. The unique bonding and radical character are confirmed by EPR spectroscopy, XANES, and quantum chemical calculations, showing spin delocalization across the M_2_P_2_ core and partial M(III) character. The *cyclo-*P_5_ ring of pentaphosphaferrocene plays an important role as a redox active P-based ligand with multidentate coordination ability. Owing to multidenticity, the {Cp_2_M}_2_{(η^4^-P_5_)FeCp*}_2_ moiety polymerizes with a potassium cation. The polymeric chain could be easily broken by extraction if potassium cations are surrounded by the cryptand. Such radical coordination polymers could have potential applications in switchable charge transport materials.

## Author contributions

X. S. and R. Y. synthesized complexes 1-M and 2-M and characterized them. X. S. conducted X-ray experiments. A. H. measured and simulated the EPR spectra and performed the theoretical calculations. S. D. synthesized and characterized complex 1-Zr′. J. G. performed the XANES measurements. P. W. R., M. S. and T. V. proposed the idea, supervised the work and interpreted the results. All authors contributed to the preparation of the manuscript.

## Conflicts of interest

There are no conflicts to declare.

## Supplementary Material

SC-OLF-D6SC04762A-s001

SC-OLF-D6SC04762A-s002

SC-OLF-D6SC04762A-s003

## Data Availability

CCDC 2497198–2497202 contain the supplementary crystallographic data for this paper.^57*a*–*e*^ IR, EPR, UV-VIS, NMR, XANES, and EA data for this paper are available at radar4chem [https://radar.products.fiz-karlsruhe.de/] at https://doi.org/10.22000/aq4yu1zg6zttxeay. Supplementary information (SI): synthesis and characterization, X-ray crystallography, IR, UV-Vis, EPR spectra, and NMR spectra, X-ray absorptions spectroscopy, and quantum chemical calculations. See DOI: https://doi.org/10.1039/d6sc04762a.
