# Supplementary material for: One-dimensional anionic radical chains based on M2P2 rings (M = Ti, Zr, Hf)
Source: Chem Sci. 2026 Jul 30;17(36):17403–8. doi: 10.1039/d6sc04762a (PMC13419976; doi:10.1039/d6sc04762a)
Supplement: Supplementary file 1 [file SC-017-D6SC04762A-s001.pdf]

Supplementary Information (SI) for Chemical Science.
This journal is © The Royal Society of Chemistry 2026

# SUPPORTING INFORMATION

Xiaofei Sun,#[a] Ravi Yadav,#[a,b], Alexander Hinz,[a] Jörg Göttlicher,[c] Tonya Vitova,[d] Sabrina Dinauer,[e] Manfred Scheer,[e] Peter W. Roesky*[a,f]

[a] Institut für Anorganische Chemie (AOC), Engesserstr. 15, 76131 Karlsruhe, Germany.

[b] Department of Chemistry, Indian Institute of Technology Roorkee, Roorkee, Uttarakhand 247667, India.

[c] Institut für Photonenforschung und Synchrotronstrahlung (IPS), Hermann-von-Helmholtz-Platz 1, 76344 Eggenstein-Leopoldshafen, Germany.

[d] Institut für Nukleare Entsorgung (INE), Hermann-von-Helmholtz-Platz 1, 76344 Eggenstein-Leopoldshafen, Germany.

[e] Institut für Anorganische Chemie, Universität Regensburg, 93040 Regensburg, Germany.

[f] Institute for Nanotechnology, Karlsruhe Institute of Technology (KIT), Kaiserstr. 12, 76131 Karlsruhe, Germany.

## Table of Contents

I. Synthesis and characterization ........ S2
- I.1 General procedures ........ S2
- I.2 Synthesis of **1-Ti**, **1-Zr**, **1-Zr’** and **1-Hf** ........ S3
- I.3 Synthesis of **2-Ti**, **2-Zr** and **2-Hf** ........ S6

II. X-ray crystallography ........ S8
- II.1 General methods ........ S8
- II.2. Summary of crystal data ........ S10
- II.3 Crystal structures ........ S12

III. IR spectra ........ S19

IV. UV-Vis spectra ........ S22

V. EPR spectra ........ S24

VI. NMR spectra ........ S28

VII. X-ray absorptions spectroscopy ........ S30

VIII. Quantum Chemical Calculations ........ S31

IX. References ........ S47

## I. Synthesis and characterization

### I.1 General procedures

All air- and moisture-sensitive manipulations were performed under dry $N_2$ or Ar atmosphere using standard Schlenk techniques or in an argon-filled MBraun glovebox, unless otherwise stated. $Et_2O$, *n*-pentane, and toluene were dried using an MBraun solvent purification system (SPS-800) and degassed. THF was distilled under nitrogen from potassium benzophenone ketyl. [Cp*Fe($\eta^5$-$P_5$)] was prepared according to the literature procedures.[1] All other chemicals were obtained from commercial sources and used without further purification.

NMR spectra were recorded on Bruker spectrometers (Avance Neo 300 MHz, Avance Neo 400 MHz or Avance III 400 MHz). Chemical shifts are referenced internally using signals of the residual protio solvent ($^1$H) and are reported relative to tetramethylsilane.

Elemental analyses were carried out with an Elementar vario MICRO cube.

Infrared (IR) spectra were recorded in the region 4000–400 $cm^{-1}$ on a Bruker Tensor 37 FTIR spectrometer equipped with a room temperature DLaTGS detector, a diamond attenuated total reflection (ATR) unit and a nitrogen-flushed chamber. In terms of their intensity, the signals were classified into different categories (vs = very strong, s = strong, m = medium, w = weak, and sh = shoulder).

UV-vis spectra were measured in solution using a Mettler-Toledo Spektralphotometer UV7 and quartz cuvettes (d = 1 cm). The sample was measured relative to the pure solvent, dry and degassed THF.

EPR spectra of **1-M** and **2-Zr** were recorded on a Bruker EMXplus X-band spectrometer (microwave frequency: 9.5 GHz). EPR spectra of **2-Ti** and **2-Hf** were recorded on a BRUKER EMX nano X-band spectrometer. EPR spectrum of **1-Zr’** was recorded on a MiniScope MS400 device with a frequency of 9.44 GHz. The spectra were simulated using the MATLAB/easyspin package.[2]

ESI mass spectra were recorded using a ThermoQuest Finnigan TSQ 7000 spectrometer by the analytical department of the University of Regensburg. All compounds were dissolved in the corresponding solvent under inert atmosphere. According to the mass/charge (m/z) ratio and the corresponding isotope pattern the observed fragments were assigned.

### I.2 Synthesis of 1-Ti, 1-Zr, 1-Zr' and 1-Hf

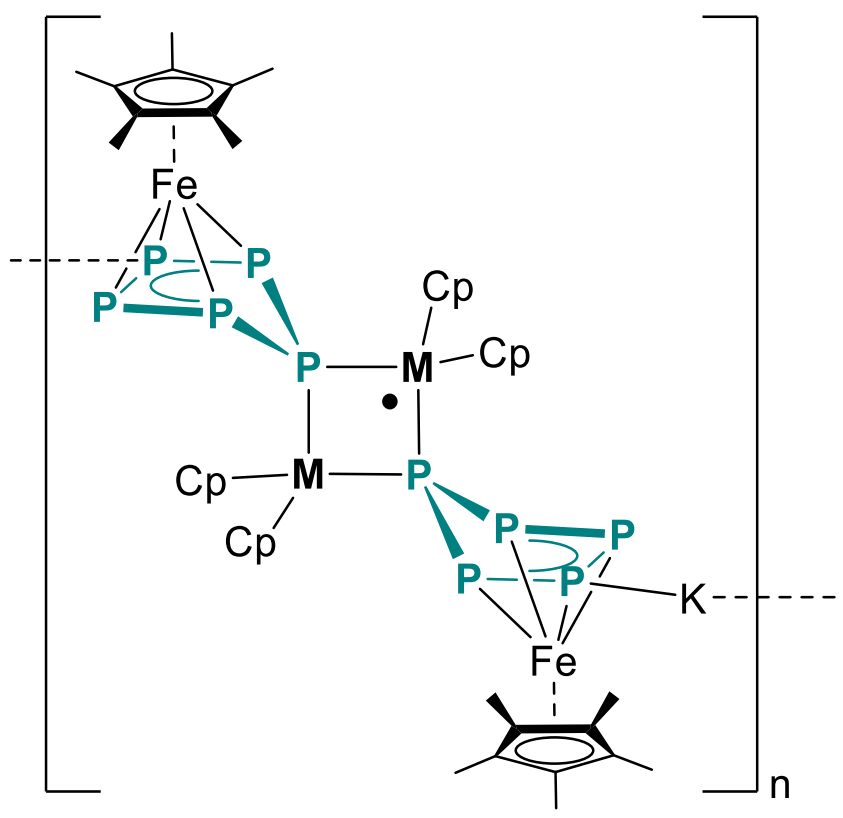

**M** = Ti (**1-Ti**), Zr (**1-Zr**), Hf (**1-Hf**)

To a J. Young Schlenk containing [Cp*Fe($\eta^5$-$P_5$)] (86.5 mg, 0.25 mmol), [$Cp_2MCl_2$] (62.2 mg for Ti, 73.1 mg for Zr, 94.9 mg for Hf, 0.25 mmol) (M = Ti, Zr, Hf) and K (29.3 mg, 0.75 mmol) was added THF (*ca.* 10 mL), the reaction mixture was sonicated at room temperature for 1 h until the green solution begun to turn purple (Zr) or dark brown (Ti, Hf). The reaction mixture was then stirred at room temperature for 48 h. After filtration, the THF solution was concentrated. Single crystals were obtained from diffusion of *n*-pentane into the THF solution over the course of two weeks.

**1-Ti:** Crystalline yield: 80 mg, 57%.

Anal. Calcd. For $C_{40}H_{50}Fe_2KP_{10}Ti_2$ (0.5 $C_4H_8O$) (1123.15 g/mol): C 44.91; H 4.85. Found: C 44.84; H 4.38.

**IR (ATR):** $\tilde{\nu}$ (cm$^{-1}$) = 3061 (w), 3047 (vw), 2963 (w), 2950 (w), 2887 (m), 2846 (w), 2185 (vw), 2164 (vw), 1990 (vw), 1982 (vw), 1474 (w), 1433 (m), 1367 (vs), 1241 (vw), 1126 (vw), 1060 (w), 1011 (s), 909 (m), 827 (s), 796 (vs), 587 (vw), 440 (w), 423 (vw).

Evans-NMR (THF-$d_8$, 298 K): $\mu_{eff}$ = 1.75 $\mu_B$ corresponding to 1.02 unpaired electrons.

UV-vis (THF): $\lambda_{max}$ [nm] ($\varepsilon$[L mol$^{-1}$ cm$^{-1}$]) = 465 (93200), 628 (23707).**1-Zr:** Crystalline yield: 74 mg, 49%.

Anal. Calcd. For $C_{40}H_{50}Fe_2KP_{10}Zr_2$ (0.5 $C_4H_8O$) (1209.87 g/mol): C 41.70; H 4.50. Found: C 41.36; H 4.42.

**IR (ATR):** $\tilde{\nu}$ (cm$^{-1}$) = 3063 (m), 2965 (s), 2885 (vs), 2867 (vs), 2166 (vw) 1476 (m), 1443 (m), 1423 (m), 1367 (s), 1288 (vw), 1265 (vw), 1173 (vw), 1118 (w), 1048 (vs), 1023 (s), 1005 (s), 892 (m), 810 (s), 786 (vs), 733 (w), 661 (vw), 458 (w), 438 (w).

$^1$H NMR (THF-$d_8$, 293 K): δ [ppm] 3.64-3.60 (m, thf), 1.80-1.75 (m, thf), 0.42 (br, ω½ = 320 Hz, $C_5Me_5$).

For the Cp ligands no signal is detectable between +100 and -100 ppm.

Evans-NMR (THF-$d_8$, 293 K): $\mu_{eff}$ = 1.64 $\mu_B$ corresponding to 0.92 unpaired electrons.

UV-vis (THF): $\lambda_{max}$ [nm] ($\varepsilon$[L mol$^{-1}$ cm$^{-1}$]) = 528 (18220), 670 (7619).

**1-Hf:** Crystalline yield: 81 mg, 47%.

Anal. Calcd. For $C_{40}H_{50}Fe_2KP_{10}Hf_2$ (0.5 $C_4H_8O$) (1384.40 g/mol): C 36.44; H 3.93. Found: C 36.98; H 3.75.

**IR (ATR):** $\tilde{\nu}$ (cm$^{-1}$) = 3080 (vw), 3055 (w), 2979 (vw), 2961 (w), 2946 (w), 2887 (m), 2709 (vw), 2324 (vw), 2189 (vw), 2166 (vw), 2117 (vw), 2076 (vw), 1986 (vw), 1474 (w), 1425 (m), 1367 (s), 1116 (vw), 1066 (w), 1007 (s), 888 (w), 819 (s), 790 (vs), 587 (vw), 501 (vw), 452 (w), 440 (w).

Evans-NMR (THF-d$_8$, 298 K): $\mu_{eff}$ = 1.78 $\mu_B$ corresponding to 1.04 unpaired electrons.

UV-vis (THF): $\lambda_{max}$ [nm] ($\varepsilon$[L mol$^{-1}$ cm$^{-1}$]) = 495 (32430), 648 (5812).

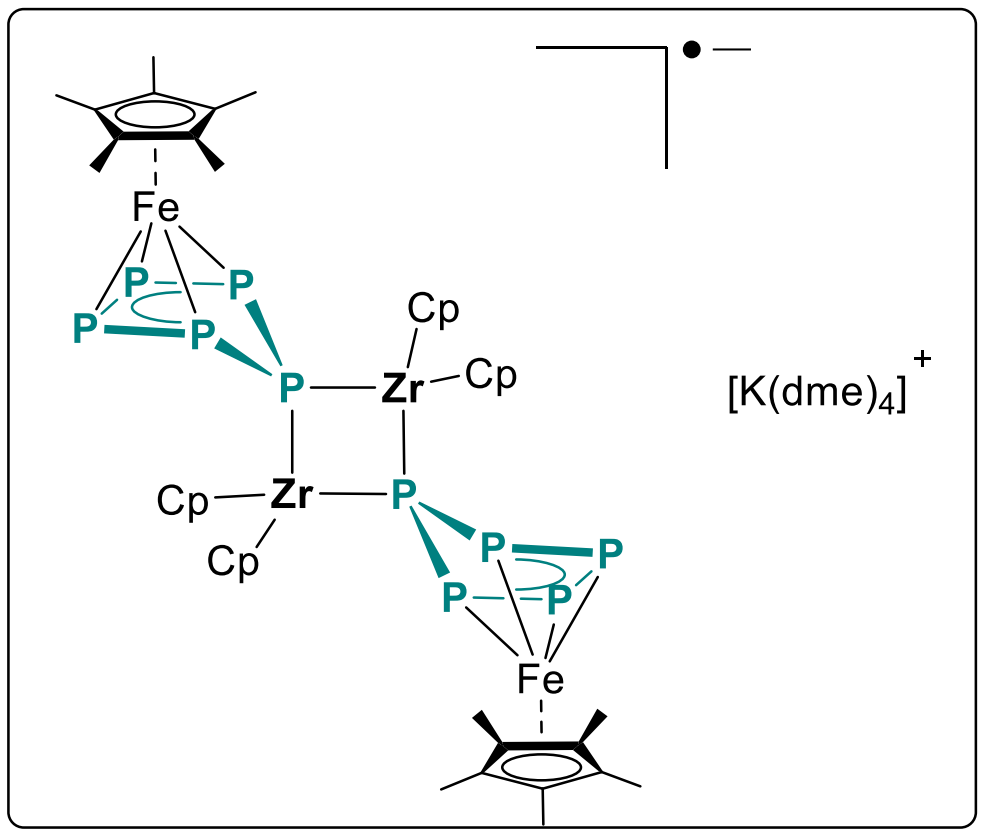


**1-Zr'**: A solution of [$Cp_2ZrCl_2$] (87.4 mg, 0.299 mmol) in DME was added to an *in situ* generated -50°C cold solution of [Cp*Fe($\eta^4$-$P_5$)]$^{2-}$ ([Cp*Fe($\eta^5$-$P_5$)]: 103.4 mg, 0.299 mmol; $KC_8$: 101.1 mg, 0.748 mmol) in DME.* The mixture was stirred overnight and allowed to reach room temperature. Upon stirring a colour change from dark green to red occurred. The solvent was removed *in vacuo* and the residue washed with hexane (3 x 5 mL), extracted with DME, and filtered over diatomaceous earth. The volume of the solution was reduced and an excess of hexane added to isolate a dark red powder of **1-Zr'**. Layering a solution of **1-Zr'** in DME under a toluene solution gives after storage at room temperature for a few days red crystals of complex **1-Zr'**. The supernatant was decanted off, the obtained crystals were washed with toluene (3 x 10 mL) and dried *in vacuo*.

Crystalline yield: 153.4 mg, 67%.

ESI-MS (DME) negative ion: *m/z* = 1133.81 ([**1-Zr'**-2dme]$^-$, 100%), 346.93 ([Cp*Fe($\eta^5$-$P_5$)]$^-$, 5%).

*The reaction works at well at room temperature, where the isolated yield is 37% (related to [Cp*Fe($\eta^5$-$P_5$)]).

$^1$H NMR (THF-$d_8$, 293 K): δ [ppm] = 3.43 (s, DME), 3.27 (s, DME), 0.41 (br, $\omega_{1/2}$ = 200 Hz, $C_5Me_5$).

For the Cp ligands no signal is detectable between +125 and -125 ppm.

Evans-NMR (THF-$d_8$, 293 K): $\mu_{eff}$ = 1.80 $\mu_B$ corresponding to 1.06 unpaired electrons.

### I.3 Synthesis of 2-Ti, 2-Zr and 2-Hf

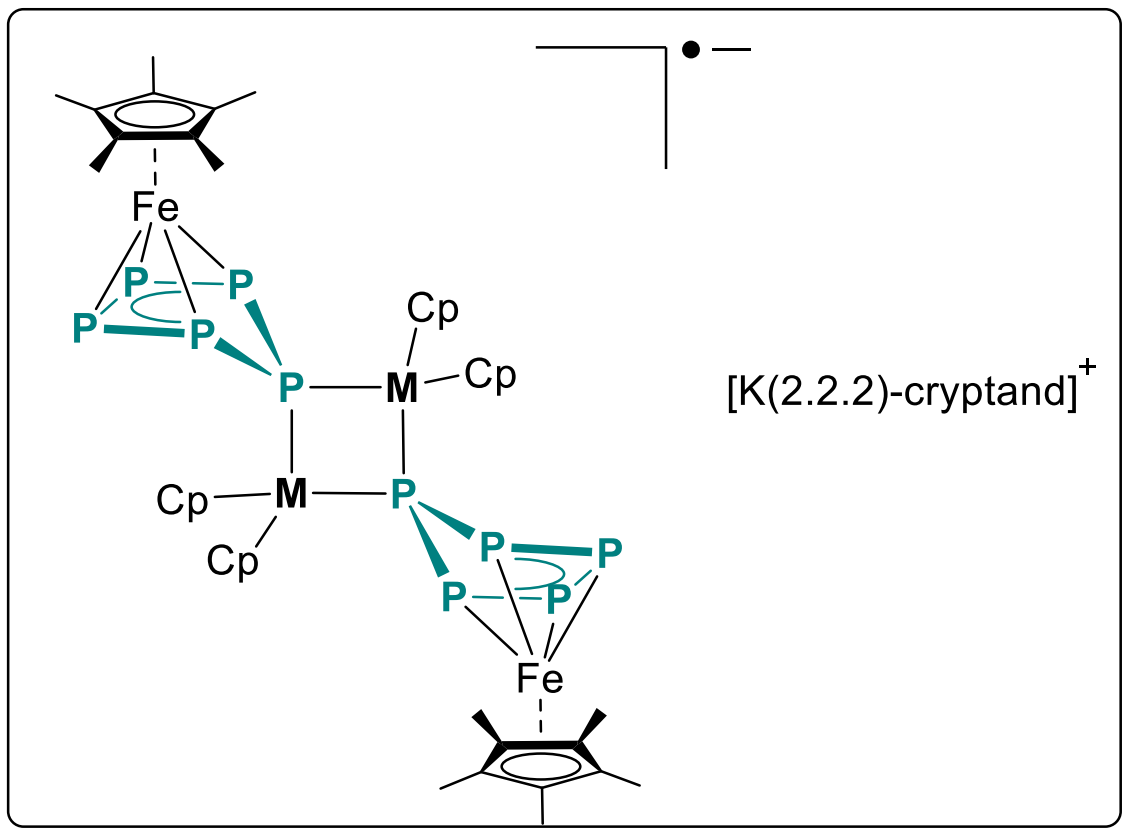


To a J. Young Schlenk flask containing **1-M** (25 mg, 0.022 mmol for Ti, 30 mg, 0.025 mmol for Zr, 30 mg, 0.022 mmol for Hf) and [2.2.2]-cryptand (12 mg, 0.032 mmol) was added THF (*ca.* 1 mL), to the solution was added *n*-pentane (*ca.* 2 mL). Single crystals of the product complexes were obtained from the THF/*n*-pentane mixture. The solution was removed with a syringe and the crystals were washed with 5 ml *n*-pentane and were dried under vacuum.

**2-Ti:** Crystalline yield: 15.2 mg, 47%.

Anal. Calcd. For $C_{58}H_{86}N_2O_6Fe_2KP_{10}Ti_2$ (1463.59 g/mol): C 47.60; H 5.92; N 1.91. Found: C 46.70; H 5.49; N 2.04.

**IR (ATR):** $\tilde{\nu}$ (cm$^{-1}$) = 3071 (w), 2959 (m), 2879 (s), 2815 (m), 2166 (w), 1984 (vw), 1529 (vw), 1474 (m), 1437 (m), 1351 (s), 1296 (w), 1255 (m), 1238 (w), 1173 (vw), 1130 (m), 1099 (vs), 1009 (s), 942 (m), 933 (m), 813 (s), 796 (s), 749 (w), 524 (vw), 436 (vw)。

**2-Zr:** Crystalline yield: 16.7 mg, 42%.

Anal. Calcd. For $C_{58}H_{86}N_2O_6Fe_2KP_{10}Zr_2$ (0.5 $C_5H_8O$) (1586.36 g/mol): C 45.43; H 5.72; N 1.77. Found: C 45.70; H 5.71; N 1.70.

**IR (ATR):** $\tilde{\nu}$ (cm$^{-1}$) = 3067 (w), 2959 (m), 2879 (vs), 2813 (m), 2711 (vw), 2166 (vw), 1474 (m), 1441 (m), 1353 (vs), 1294 (m), 1257 (m), 1238 (w), 1173 (vw), 1130 (m), 1099 (vs), 1021 (m), 1007 (m), 946 (m), 931 (m), 802 (vs), 733(w), 692 (vw), 579 (vw), 524 (vw), 458 (w), 440 (vw).

$^1$H NMR (THF-d$_8$, 293 K): δ [ppm] 3.64 (s, crypt), 2.60 (s, crypt), 0.40 (br, $C_5Me_5$).

For the Cp ligands no signal is detectable between +100 and -100 ppm.

**2-Hf:** Crystalline yield: 16.3 mg, 43%.

Anal. Calcd. For $C_{58}H_{86}N_2O_6Fe_2KP_{10}Hf_2$ (1724.84 g/mol): C 40.39; H 5.03; N 1.62. Found: C 39.67; H 4.62; N 1.66.

**IR (ATR):** $\tilde{\nu}$ (cm$^{-1}$) = 3071 (w), 2959 (m), 2879 (s), 2705 (vw), 2324 (vw), 2185 (vw), 2166 (vw), 2146 (vw), 2119 (vw), 2082 (vw), 2074 (vw), 1984 (vw), 1474 (m), 1441 (m), 1353 (m), 1298 (m), 1253 (s), 1236 (s), 1198 (vw), 1183 (vw), 1128 (m), 1099 (vs), 1023 (w), 1005 (m), 985 (m), 944 (m), 808 (s), 747 (m), 571 (vw), 524 (vw), 456 (w), 440 (w).

Measuring the magnetic moment using the Evans NMR method were unsuccessful because of insufficient solubility of **2-M** in suitable deuterated solvents, leading to unreliable chemical shift separation.

## II. X-ray crystallography

### II.1 General methods

Suitable crystals for the X-ray analysis of all compounds were obtained as described above. A suitable crystal was covered in mineral oil (Aldrich) and mounted on a glass fibre. The crystal was transferred directly to the cold stream of a STOE StadiVari (100 K) diffractometer. All structures were solved by using the program SHELXS/T[3,4,] and Olex2.[5] The remaining non-hydrogen atoms were located from successive difference Fourier map calculations. The refinements were carried out by using full-matrix least-squares techniques on $F^2$ by using the program SHELXL.[4,5] The H-atoms were introduced into the geometrically calculated positions (SHELXL procedures) unless otherwise stated and refined riding on the corresponding parent atoms. In each case, the locations of the largest peaks in the final difference Fourier map calculations, as well as the magnitude of the residual electron densities, were of no chemical significance. Specific comments for each data set are given below. Summary of the crystal data, data collection and refinement for compounds are given in Table S1.

The X-ray diffraction experiment for **1-Zr'** was performed on a Super Nova diffractometer with a Titan2S detector applying Cu-$K_\alpha$ radiation with a HyPix-Arc 150 detector using Cu-K$\alpha$ radiation from a rotating anode. The measurement was performed at 123 K. Data collection and reduction were performed with CrysAlispro (Version 1.171.4148a). A numeric absorption correction based on gaussian integration over a multifaceted crystal model was used. The structure was solved by direct methods with ShelXT and Olex2 and refined by full-matrix least-squares method against $F^2$ in anisotropic approximation using ShelXL.[10] All non-hydrogen atoms were refined anisotropically. Hydrogen atoms were refined in calculated positions riding on pivot atom model.

Crystallographic data for the structures reported in this paper have been deposited with the Cambridge Crystallographic Data Centre as a supplementary publication no. 2497198-2497202 and 2500619-2500620. Copies of the data can be obtained free of charge on application to CCDC, 12 Union Road, Cambridge CB21EZ, UK (fax: (+(44)1223-336-033; email: deposit@ccdc.cam.ac.uk).

<u>The following special comments apply to the models of the structures:</u>

In the crystal structure of complex **1-Zr**, one co-crystallized THF molecule (O3, C29-C32) is disordered over two positions with an occupancy ratio of 0.86/0.14.

In the crystal structure of complex **2-Ti**, two $C_5H_5$ groups (C11-C15 and C16-C20) are disordered over two positions with an occupancy ratio of 0.53/0.43 and 0.52/0.48, respectively.

The crystal of **2-Zr** consists of two twin domains. It was refined with the twin domain ratio of 0.56/0.44. In the crystal structure of complex **2-Zr**, one $C_5H_5$ group (C11-C15) is disordered over two positions with an occupancy ratio of 0.57/0.43.

In the crystal structure of complex **2-Hf**, two $C_5H_5$ groups (C11-C15 and C16-C20) are disordered over two positions with an occupancy ratio of 0.54/0.46 and 0.52/0.48, respectively.

In the crystal structure of complex **1-Zr’**, one Cp* and three Cp ligands and the dme molecules are disordered over two positions (occupancy of 0.4865 and 0.5135).

## II.2. Summary of crystal data

**Table S1.** Crystal data, data collection and refinement for compounds **1** and **2**.

| Compound | 1-Ti | 1-Zr | 1-Hf | 1-Zr' |
|---|---|---|---|---|
| | | | | |
| Formula | $C_{64}H_{98}Fe_2KO_6P_{10}Ti_2$ | $C_{64}H_{98}Fe_2KO_6P_{10}Zr_2$ | $C_{64}H_{98}Fe_2Hf_2KO_6P_{10}$ | $C_{56}H_{90}Fe_2KO_8P_{10}Zr_2$ |
| $D_{calc.}$/ g cm$^{-3}$ | 1.420 | 1.470 | 1.629 | 1.476 |
| $\mu$/mm$^{-1}$ | 0.948 | 0.996 | 3.566 | 8.823 |
| Formula Weight | 1519.72 | 1606.36 | 1780.90 | 1534.21 |
| Colour | yellow | violet | yellow | dark red |
| Shape | plate-shaped | plate-shaped | plate-shaped | block-shaped |
| Size/mm$^3$ | 0.18×0.12×0.05 | 0.21×0.14×0.04 | 0.25×0.17×0.04 | 0.51×0.16×0.11 |
| $T$/K | 100 | 100 | 100 | 123.01(10) |
| Crystal System | monoclinic | monoclinic | monoclinic | monoclinic |
| Space Group | $C2/c$ | $C2/c$ | $C2/c$ | $P2_1/n$ |
| $a$/Å | 30.6602(14) | 30.8091(10) | 30.7382(13) | 16.47870(10) |
| $b$/Å | 11.8490(4) | 12.0508(5) | 12.0606(3) | 24.2741(29 |
| $c$/Å | 21.4939(10) | 21.4761(7) | 21.4664(8) | 18.2055(2) |
| $\alpha$/° | | | | |
| $\beta$/° | 114.482(4) | 114.483(2) | 114.186(3) | 108.5070(10) |
| $\gamma$/° | | | | |
| V/Å$^3$ | 7106.5(6) | 7256.6(5) | 7259.5(5) | 6905.69(11) |
| $Z$ | 4 | 4 | 4 | 4 |
| Wavelength/Å | 0.71073 | 0.71073 | 0.71073 | 1.54184 |
| Radiation type | Mo $K_\alpha$ | Mo $K_\alpha$ | Mo $K_\alpha$ | Cu $K_\alpha$ |
| $\Theta_{min}$/° | 1.867 | 1.960 | 1.838 | 3.364 |
| $\Theta_{max}$/° | 25.998 | 25.248 | 25.996 | 72.635 |
| Measured Refl's. | 23752 | 17196 | 44441 | 50641 |
| Indep't Refl's | 6968 | 6562 | 7134 | 13568 |
| Refl's I≥2 $\sigma$(I) | 5304 | 5192 | 5049 | 11322 |
| $R_{int}$ | 0.0338 | 0.0320 | 0.0687 | 0.0371 |
| GooF | 1.000 | 1.029 | 1.056 | 0.972 |
| $wR_2$ (all data) | 0.0820 | 0.0988 | 0.1307 | 0.0828 |
| $wR_2$ | 0.0763 | 0.0916 | 0.1180 | 0.0807 |
| $R_1$ (all data) | 0.0524 | 0.0551 | 0.0734 | 0.0383 |
| $R_1$ | 0.0331 | 0.0388 | 0.0471 | 0.0318 |

| **Compound** | **2-Ti** | **2-Zr** | **2-Hf** |
|---|---|---|---|
| | | | |
| Formula | $C_{62}H_{94}Fe_2KN_2O_7P_{10}Ti_2$ | $C_{62}H_{94}Fe_2KN_2O_7P_{10}Zr_2$ | $C_{62}H_{94}Fe_2KN_2O_7P_{10}Hf_2$ |
| $D_{calc.}$/ g cm$^{-3}$ | 1.465 | 1.507 | 1.680 |
| $\mu$/mm$^{-1}$ | 0.970 | 1.013 | 3.651 |
| Formula Weight | 1535.69 | 1622.33 | 1794.85 |
| Colour | yellowgreen | violet | brown |
| Shape | plate-shaped | plate-shaped | plate-shaped |
| Size/mm$^3$ | 0.16×0.12×0.06 | 0.18×0.10×0.06 | 0.35×0.22×0.1 |
| $T$/K | 100 | 100 | 100 |
| Crystal System | monoclinic | monoclinic | monoclinic |
| Space Group | $C2/c$ | $C2/c$ | $C2/c$ |
| $a$/Å | 32.951(7) | 33.429(6) | 33.435(3) |
| $b$/Å | 11.904(2) | 11.9781(12) | 11.9280(6) |
| $c$/Å | 17.766(4) | 17.856(3) | 17.800(2) |
| $\alpha$/° | | | |
| $\beta$/° | 91.96(2) | 91.606(14) | 91.712(8) |
| $\gamma$/° | | | |
| V/Å$^3$ | 6965(2) | 7147(2) | 7095.5(10) |
| $Z$ | 4 | 4 | 4 |
| Wavelength/Å | 0.71073 | 0.71073 | 0.71073 |
| Radiation type | Mo $K_\alpha$ | Mo $K_\alpha$ | Mo $K_\alpha$ |
| $\Theta_{min}$/° | 2.139 | 2.129 | 2.13 |
| $\Theta_{max}$/° | 26.000 | 25.250 | 29.80 |
| Measured Refl's. | 20909 | 30245 | 45327 |
| Indep't Refl's | 6832 | 30245 | 7344 |
| Refl's I≥2 $\sigma$(I) | 4200 | 18732 | 5945 |
| $R_{int}$ | 0.0515 | | 0.0475 |
| GooF | 0.969 | 0.950 | 1.021 |
| $wR_2$ (all data) | 0.1282 | 0.1390 | 0.0787 |
| $wR_2$ | 0.1089 | 0.1150 | 0.0732 |
| $R_1$ (all data) | 0.0927 | 0.0945 | 0.0434 |
| $R_1$ | 0.0458 | 0.0475 | 0.0301 |

## II.3 Crystal structures

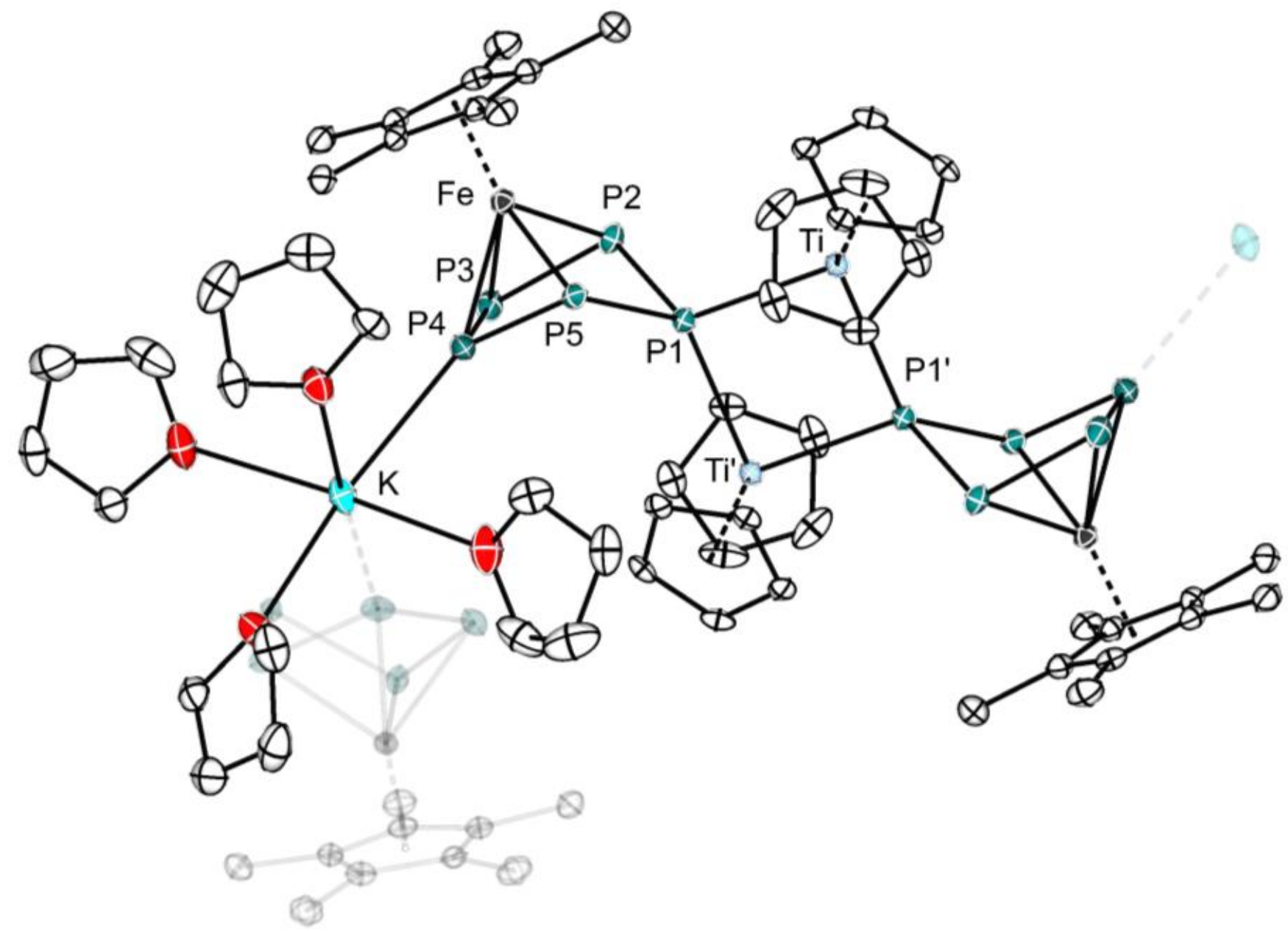


**Figure S1.** Molecular structure of the complex **1-Ti** in the solid state with thermal ellipsoids at 40% level. All hydrogen atoms are omitted for clarity. Selected bond distances [Å] and angles [°]: Ti–P1 2.5731(7), Ti–P1′ 2.6052(7), P1–P2 2.1993(8), P2–P3 2.1395(9), P3–P4 2.1523(9), P4–P5 2.1497(9), P1–P5 2.2054(8), Fe–P2 2.2688(6), Fe–P3 2.3340(7), Fe–P4 2.3232(7), Fe–P5 2.2742(6), P4–K 3.3584(8); Ti–P1–Ti′ 89.86(2), P1–Ti–P1′ 90.14(2).

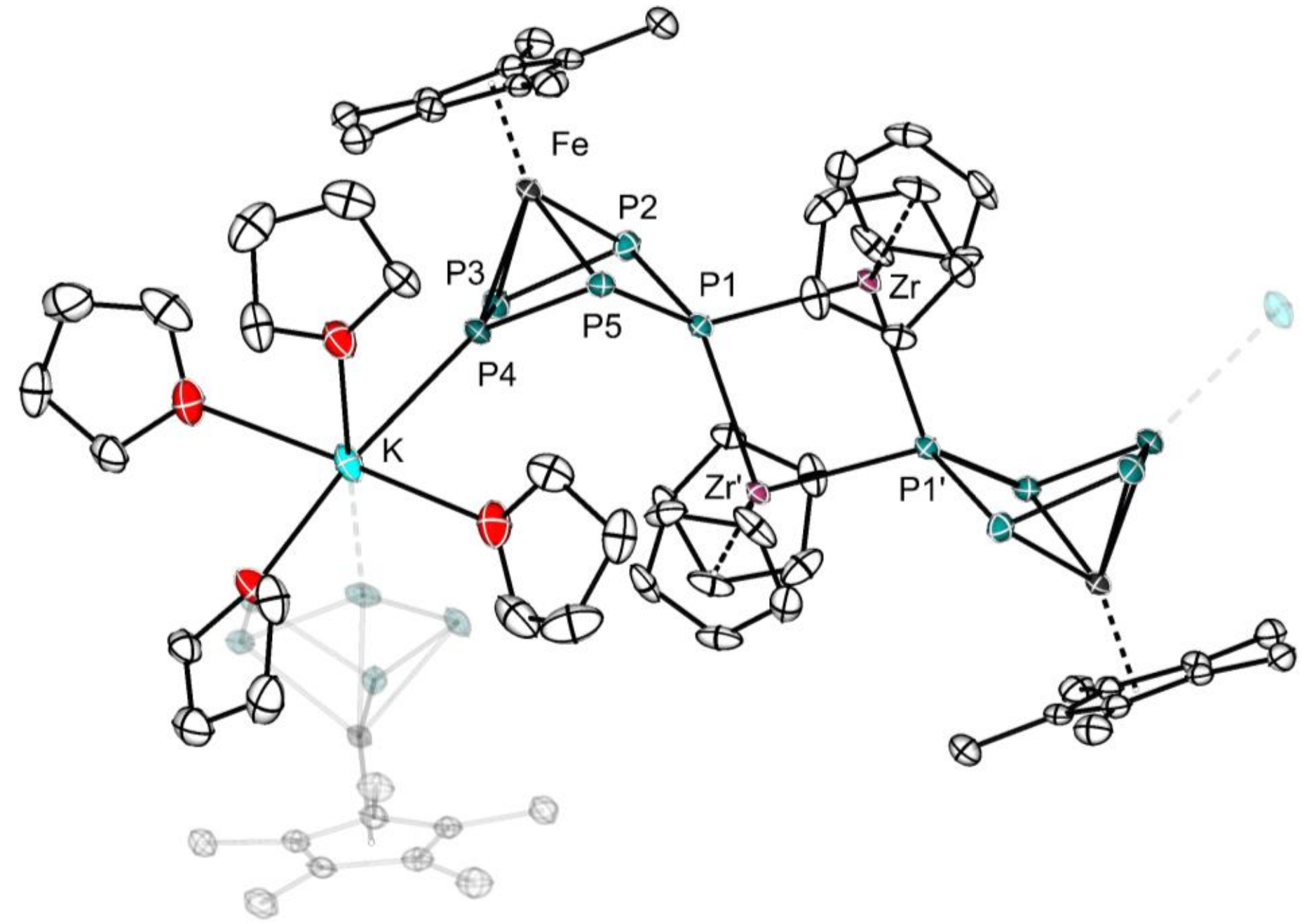


**Figure S2.** Molecular structure of the complex **1-Zr** in the solid state with thermal ellipsoids at 40% level. All hydrogen atoms are omitted for clarity. Selected bond distances [Å] and angles [°]: Zr–P1 2.6527(8), Zr – P1' 2.6782(9), P1–P2 2.1898(12), P2–P3 2.1420(13), P3–P4 2.1475(15), P4–P5 2.1514(12), P1–P5 2.1961(12), Fe–P2 2.2756(10), Fe–P3 2.3357(10), Fe–P4 2.3289(10), Fe–P5 2.2802(9), P4–K 3.3571(13); Zr–P1–Zr' 87.26(3), P1–Zr–P1' 92.74(3).

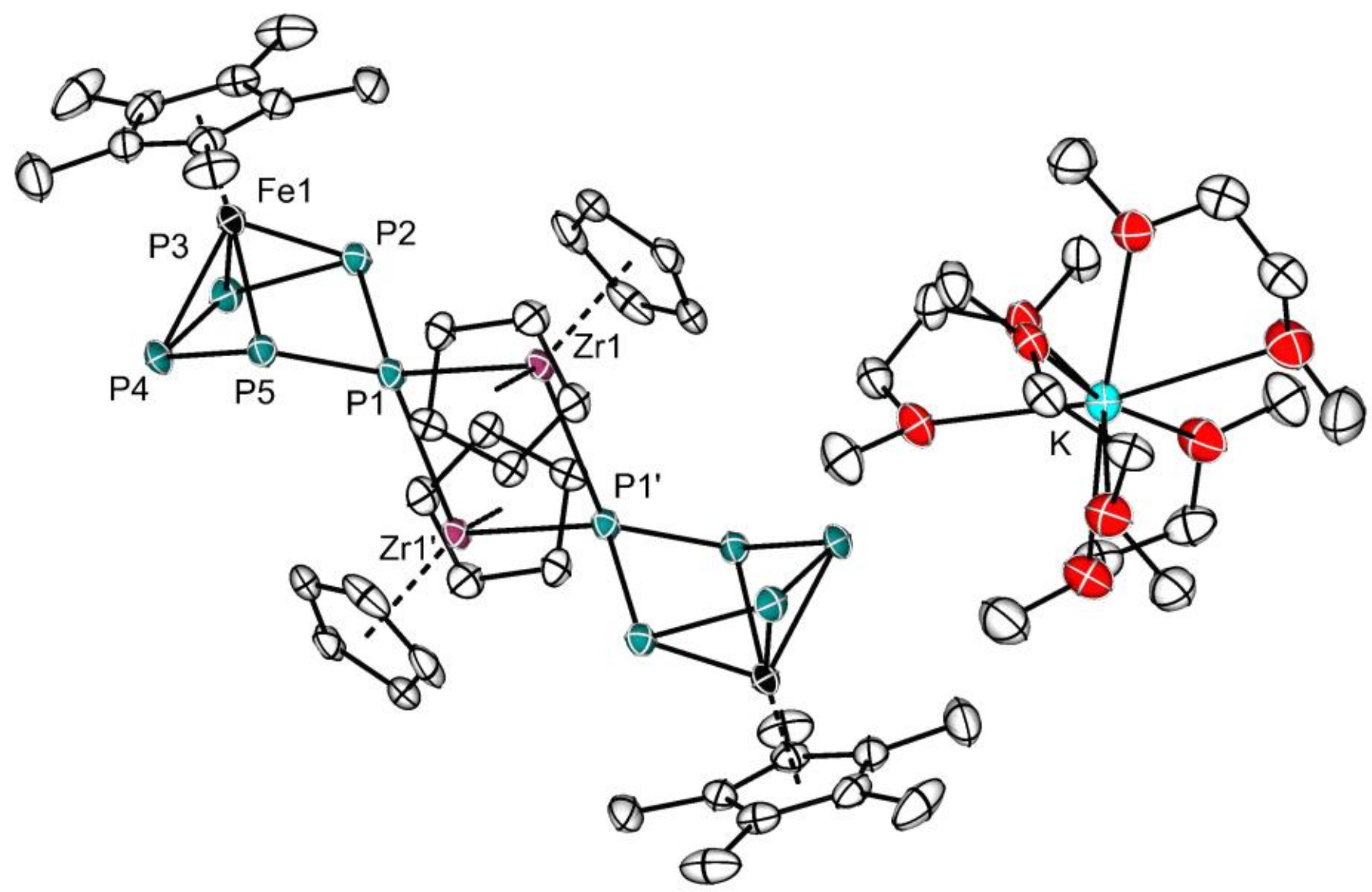


**Figure S3.** Molecular structure of the complex **1-Zr'** in the solid state with thermal ellipsoids at 40% level. All hydrogen atoms are omitted for clarity. Selected bond distances [Å] and angles [°]: Zr–P1 2.6713(6), Zr–P1' 2.6750(6), P1–P2 2.1953(8), P2–P3 2.1546(9), P3–P4 2.1534(10), P4–P5 2.1497(9), P1–P5 2.2005(8), Fe1–P2 2.2802(7), Fe–P3 2.3322(8), Fe–P4 2.3381(8), Fe–P5 2.2823(7), Zr–P1–Zr' 85.61(2), P1–Zr–P1' 94.39(2).

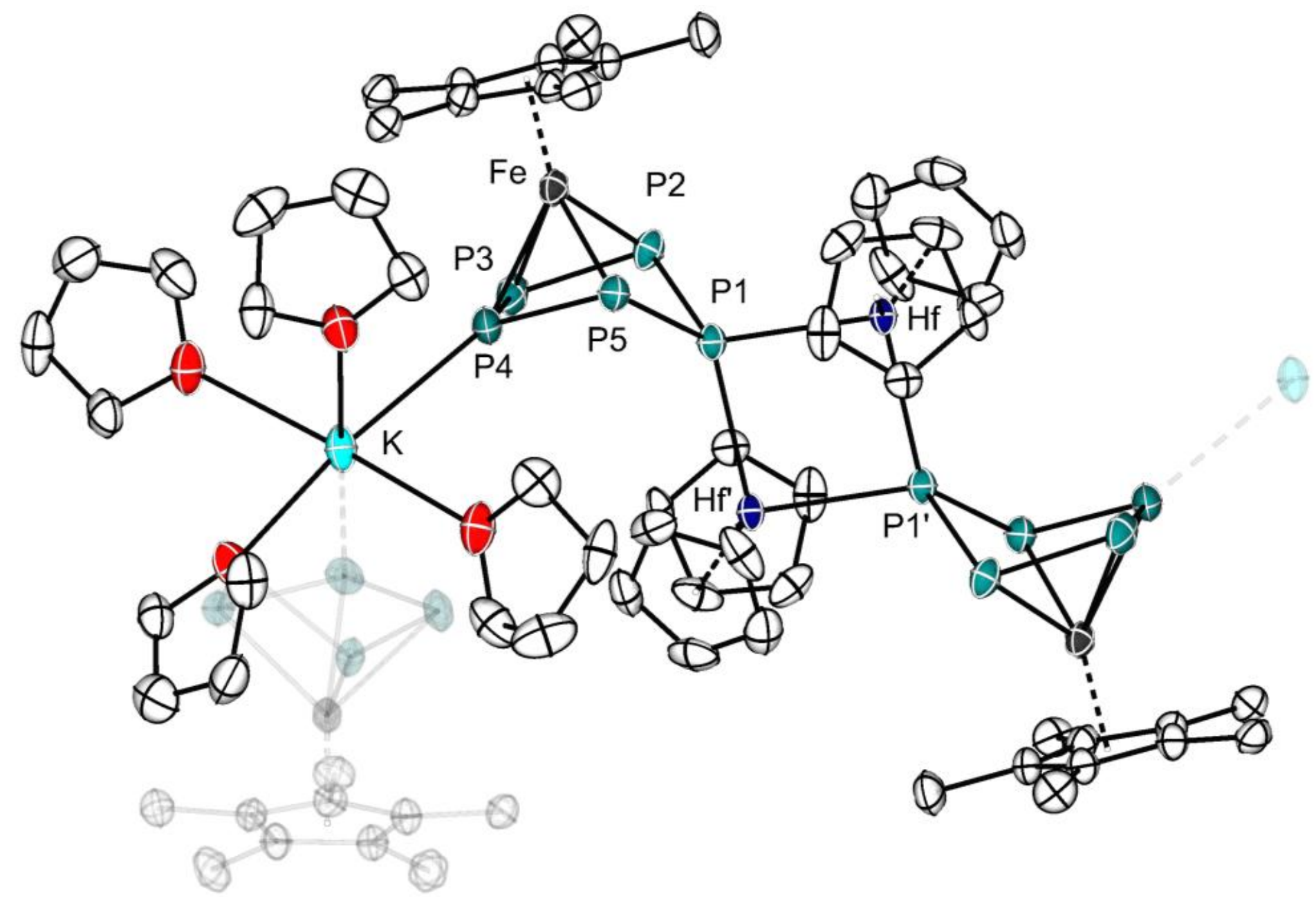


**Figure S4.** Molecular structure of the complex **1-Hf** in the solid state with thermal ellipsoids at 40% level. All hydrogen atoms are omitted for clarity. Selected bond distances [Å] and angles [°]: Hf–P1 2.6315(15), Hf –P1′ 2.648(2), P1–P2 2.190(2), P2–P3 2.140(3), P3–P4 2.149(3), P4–P5 2.153(2), P1–P5 2.203(2), Fe–P2 2.269(2), Fe–P3 2.333(2), Fe–P4 2.329(2), Fe–P5 2.275(2), P4–K 3.360(2); Hf–P1–Hf’ 87.97(5), P1–Hf–P1’ 92.03(5).

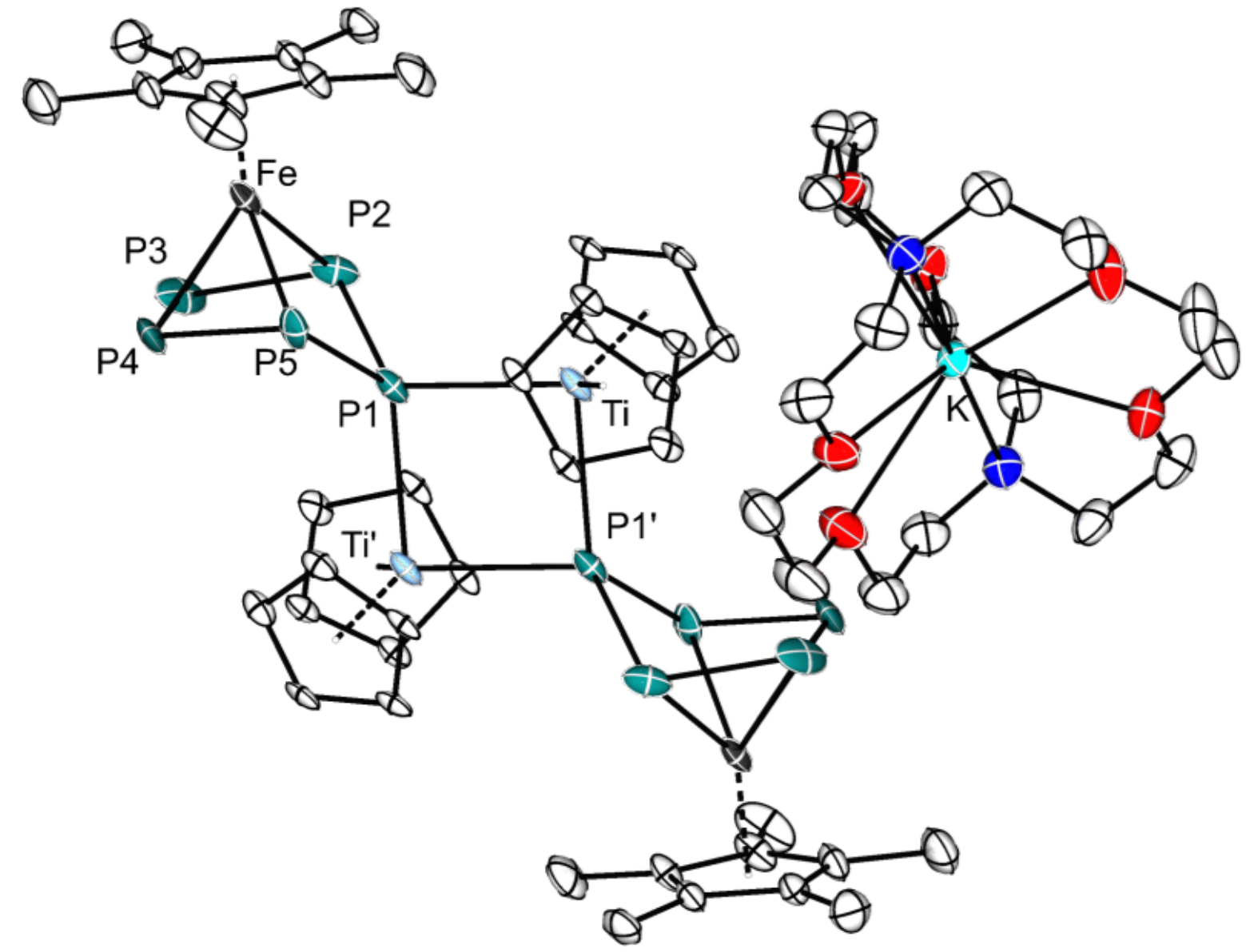


**Figure S5.** Molecular structure of the complex **2-Ti** in the solid state with thermal ellipsoids at 40% level. All hydrogen atoms and the non-coordinating THF molecule are omitted for clarity. Selected bond distances [Å] and angles [°]: Ti–P1 2.5824(4), Ti–P1' 2.5974(4), P1–P2 2.1963(15), P2–P3 2.139(2), P3–P4 2.142(2), P4–P5 2.1431(15), P1–P5 2.194(2), Fe–P2 2.2651(14), Fe–P3 2.3216(13), Fe–P4 2.3237(12), Fe–P5 2.2731(12); Ti–P1–Ti' 89.40(4), P1–Ti–P1' 90.60(4).

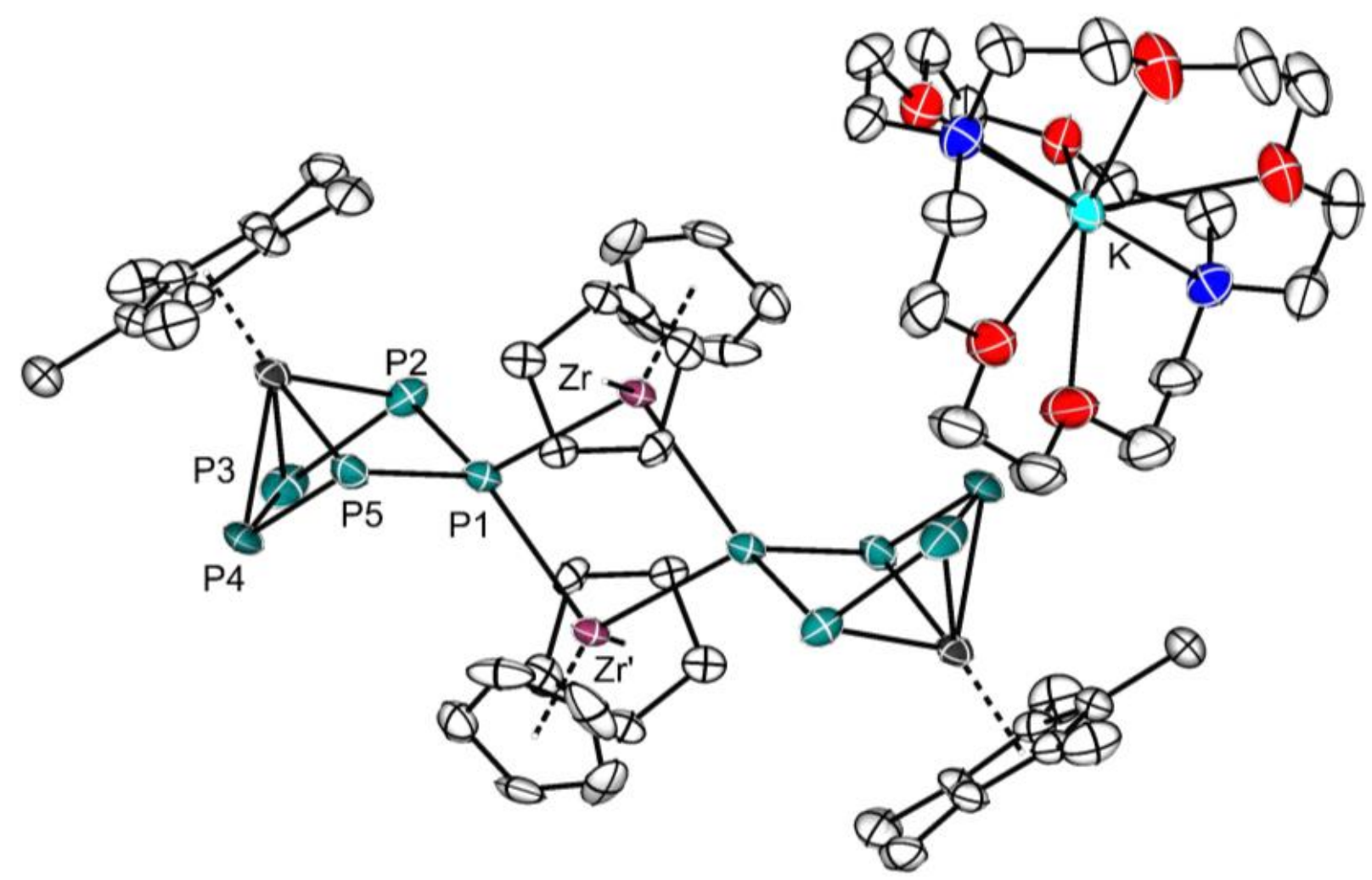


**Figure S6.** Molecular structure of the complex **2-Zr** in the solid state with thermal ellipsoids at 40% level. All hydrogen atoms and the non-coordinating THF molecule are omitted for clarity. Selected bond distances [Å] and angles [°]: Zr–P1 2.658(2), Zr –P1‘ 2.676(2), P1–P2 2.191(2), P2–P3 2.155(3), P3–P4 2.143(3), P4–P5 2.154(2), P1–P5 2.195(3), Fe–P2 2.280(2), Fe–P3 2.328(2), Fe–P4 2.333(2), Fe–P5 2.279(2); Zr–P1–Zr’ 87.22(5), P1–Zr–P1’ 92.78(5).

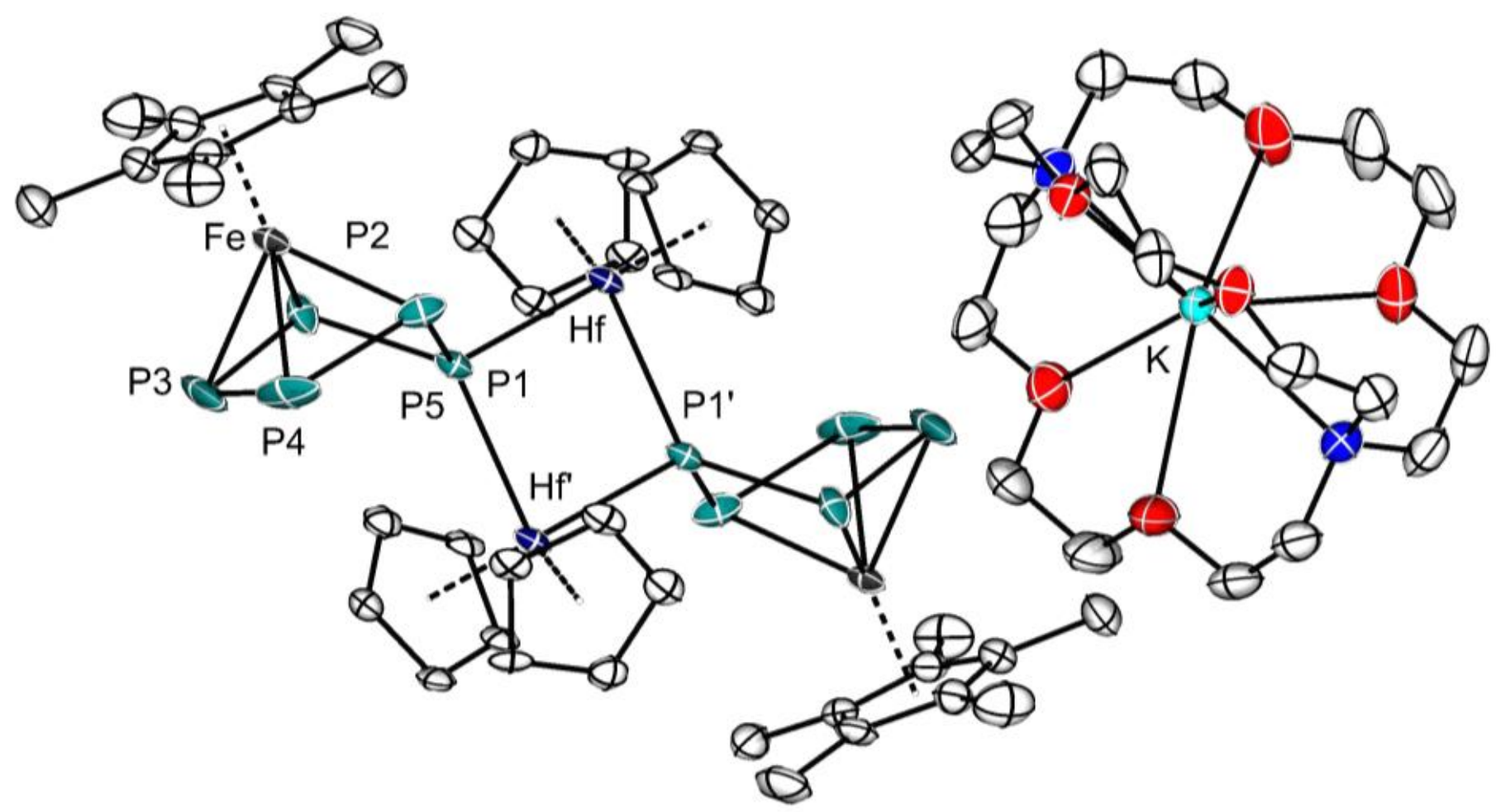


**Figure S7.** Molecular structure of the complex **2-Hf** in the solid state with thermal ellipsoids at 40% level. All hydrogen atoms and the non-coordinating THF molecule are omitted for clarity. Selected bond distances [Å] and angles [°]: Hf–P1 2.6432(10), Hf –P1‘ 2.6413(11), P1–P2 2.191(2), P2–P3 2.155(3), P3–P4 2.143(3), P4–P5 2.154(2), P1–P5 2.195(3), Fe–P2 2.280(2), Fe–P3 2.328(2), Fe–P4 2.333(2), Fe–P5 2.279(2); Hf–P1–Hf' 87.90(3), P1–Hf–P1' 92.10(3).

**Table S2.** Selected bond lengths and angles in complexes **1-M** and **2-M**.

| **Complex** | **1-Ti** | **1-Zr** | **1-Hf** | **2-Ti** | **2-Zr** | **2-Hf** | **1-Zr'** |
|---|---|---|---|---|---|---|---|
| M–P1 | 2.5730(7) | 2.6527(8) | 2.6315(15) | 2.5824(4) | 2.678(2) | 2.6432(10) | 2.6713(6) |
| M–P1' | 2.6052(7) | 2.6782(9) | 2.648(2) | 2.5974(4) | 2.676(2) | 2.6413(11) | 2.6750(6) |
| P1–M–P1' | 90.14(2) | 92.74(3) | 92.03(5) | 90.60(4) | 92.78(5) | 92.10(3) | 94.39(2). |
| M–P1–M' | 89.86(2) | 87.26(3) | 87.97(5) | 89.40(4) | 87.22(5) | 87.90(3) | 85.61(2. |

## III. IR spectra

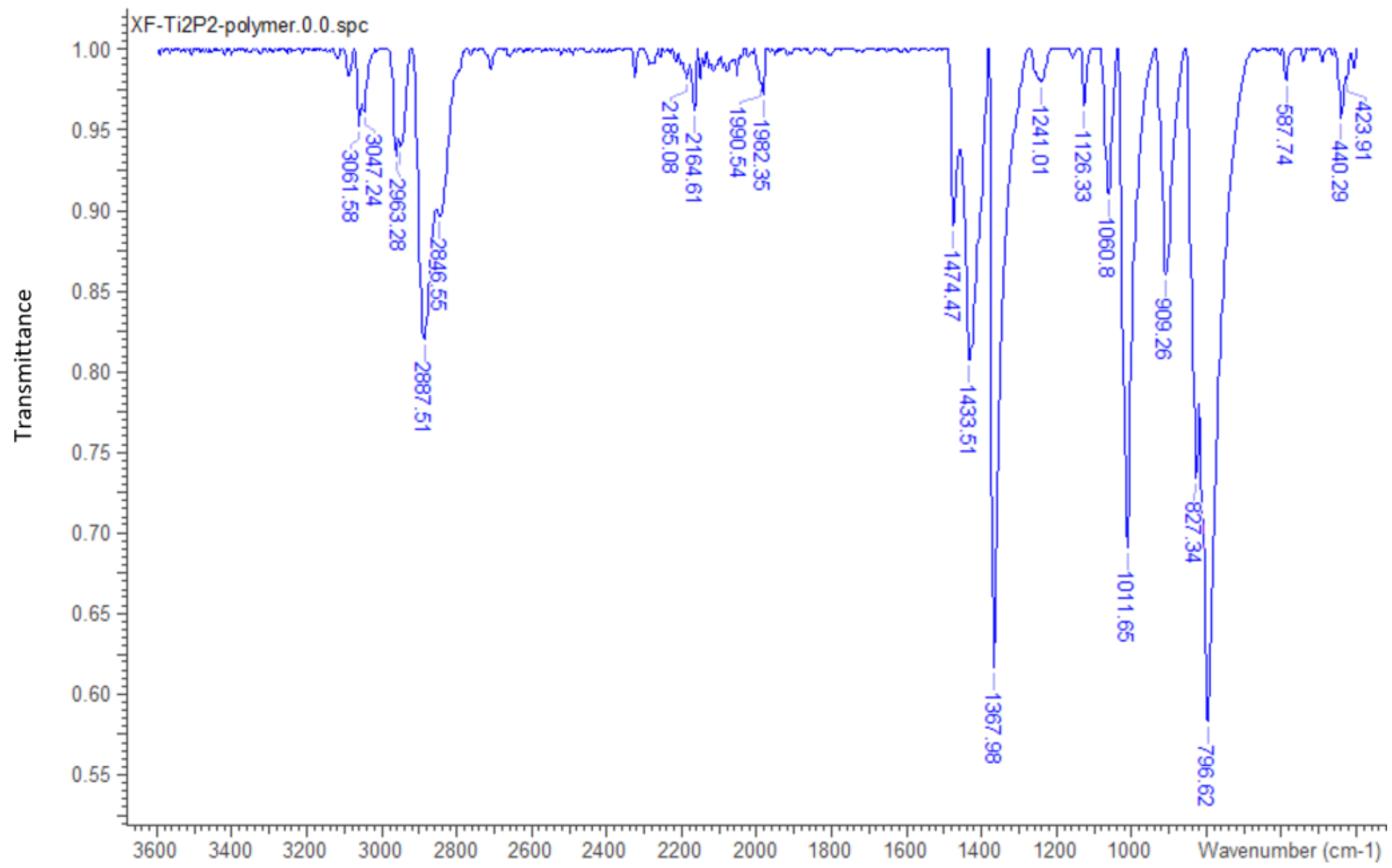


**Figure S8.** IR spectrum of complex **1-Ti**.

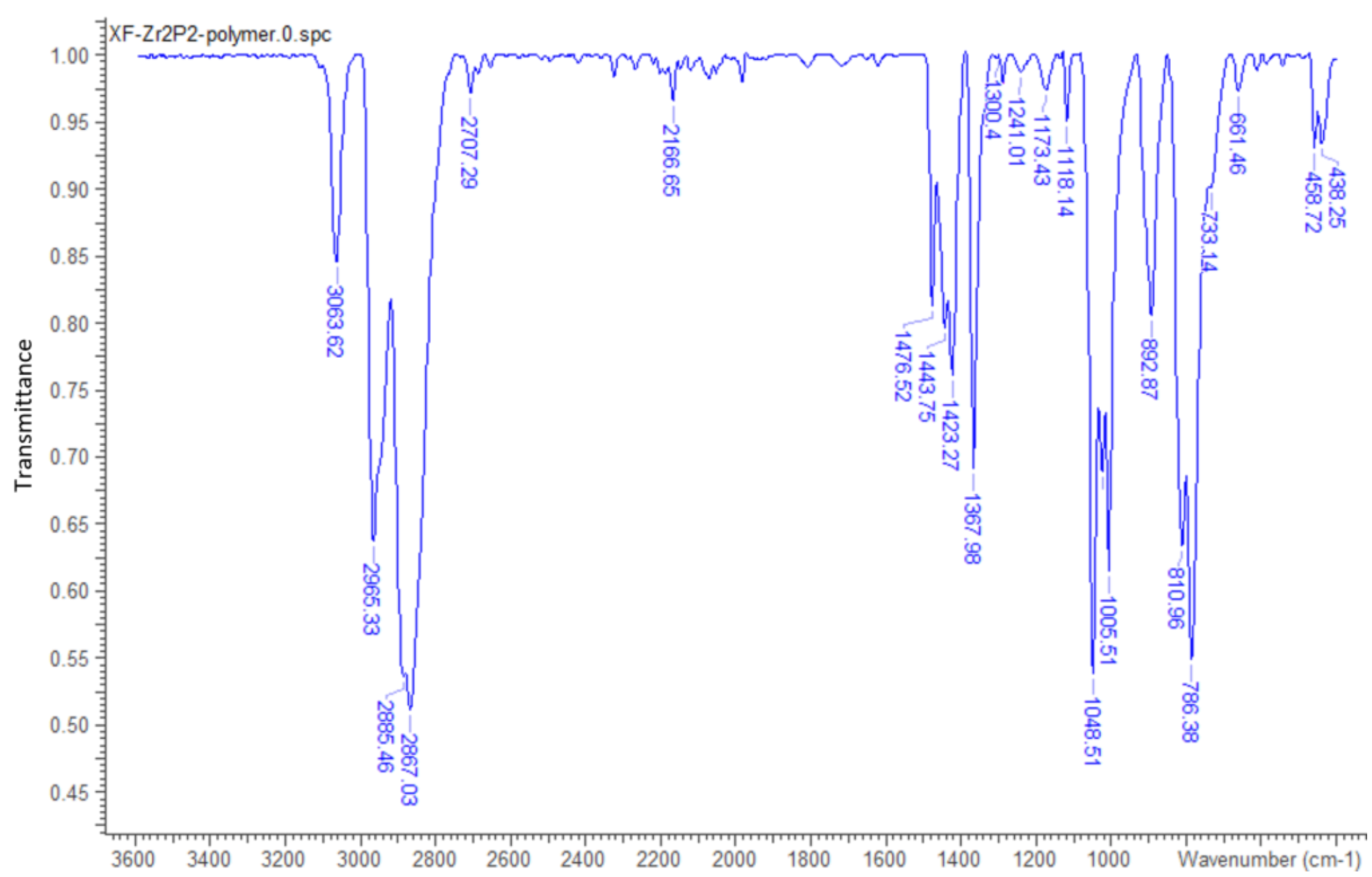


**Figure S9.** IR spectrum of complex **1-Zr**.

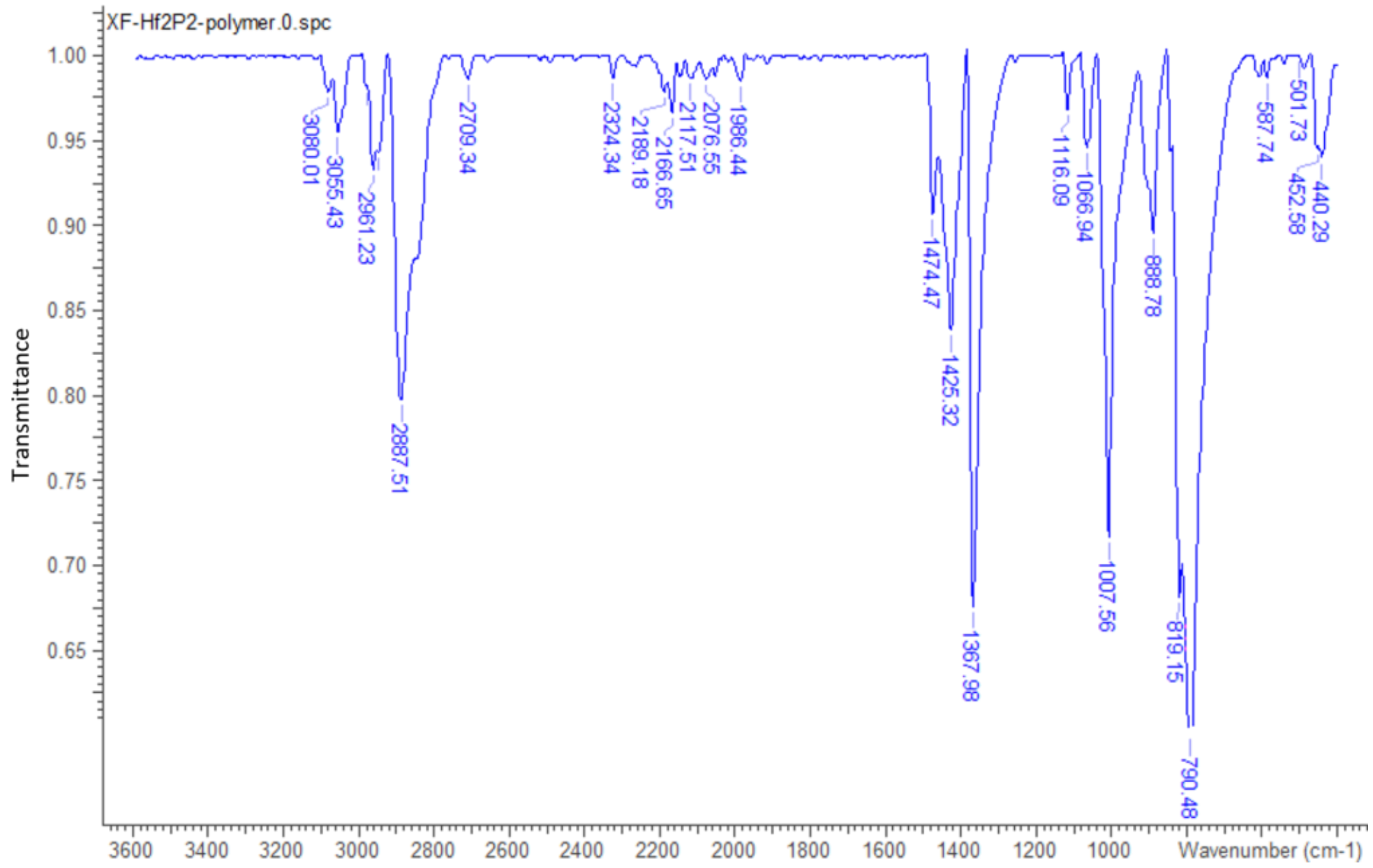


**Figure S10.** IR spectrum of complex **1-Hf**.

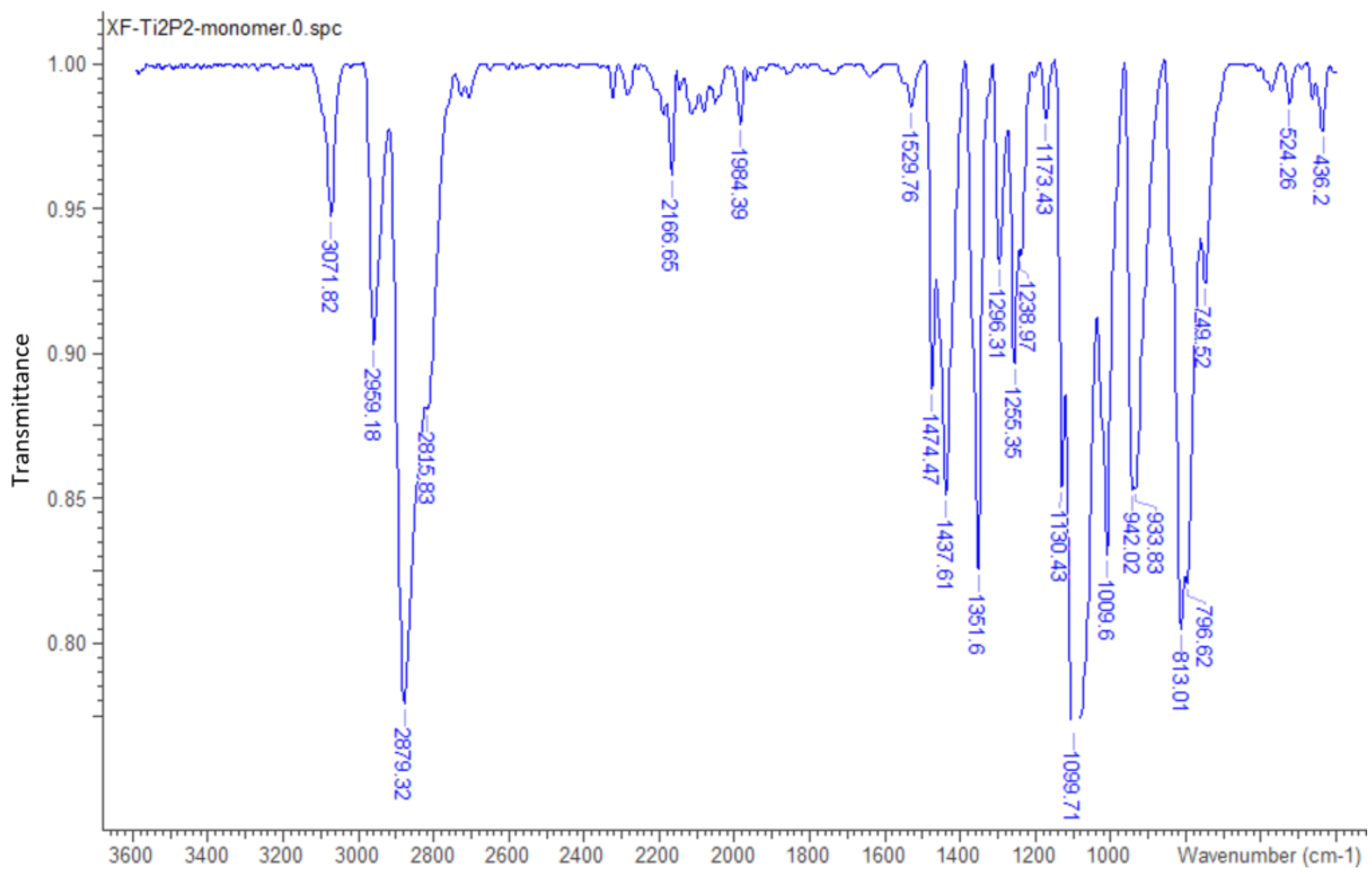


**Figure S11.** IR spectrum of complex **2-Ti**.

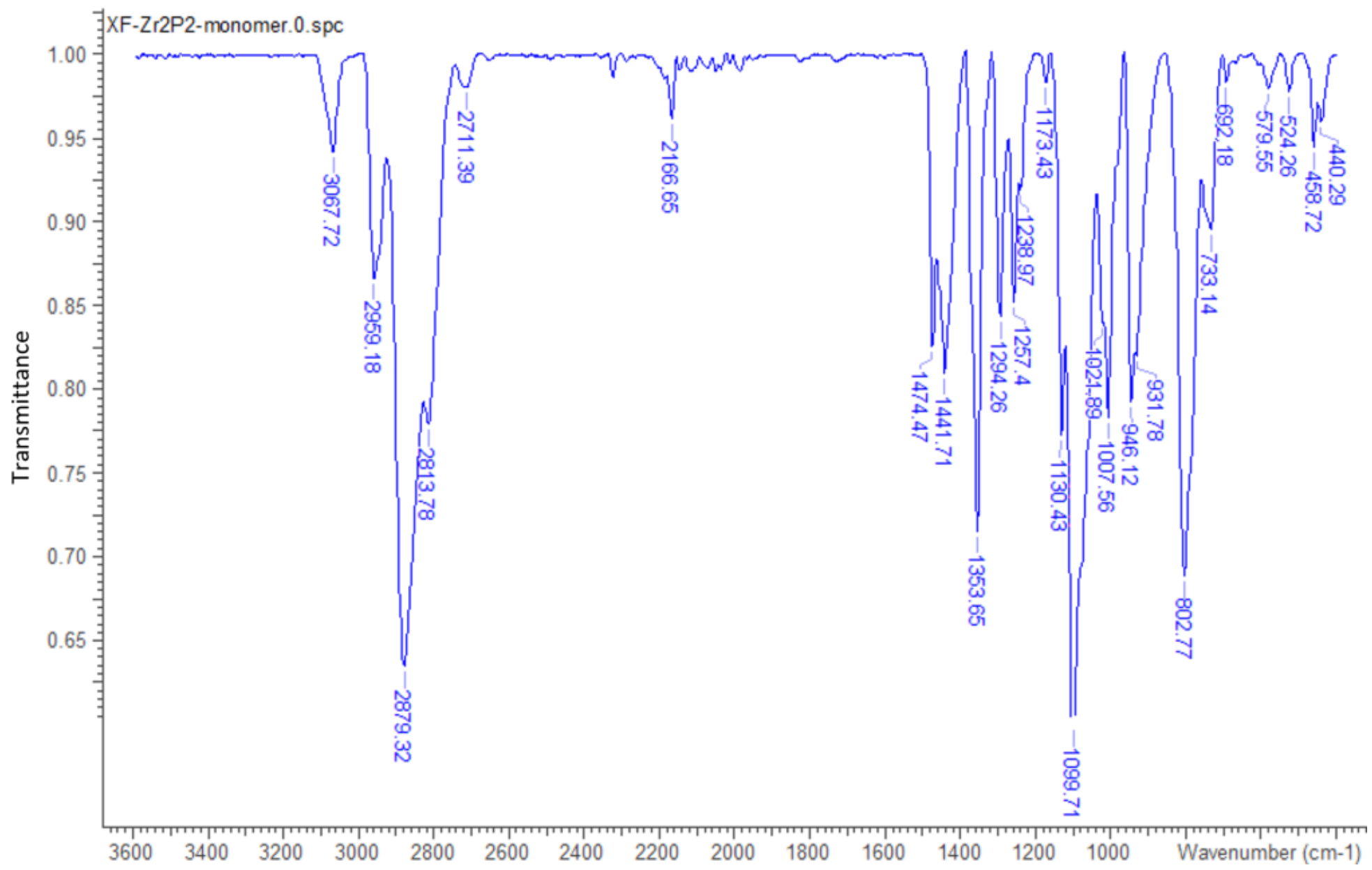


**Figure S12.** IR spectrum of complex **2-Zr**.

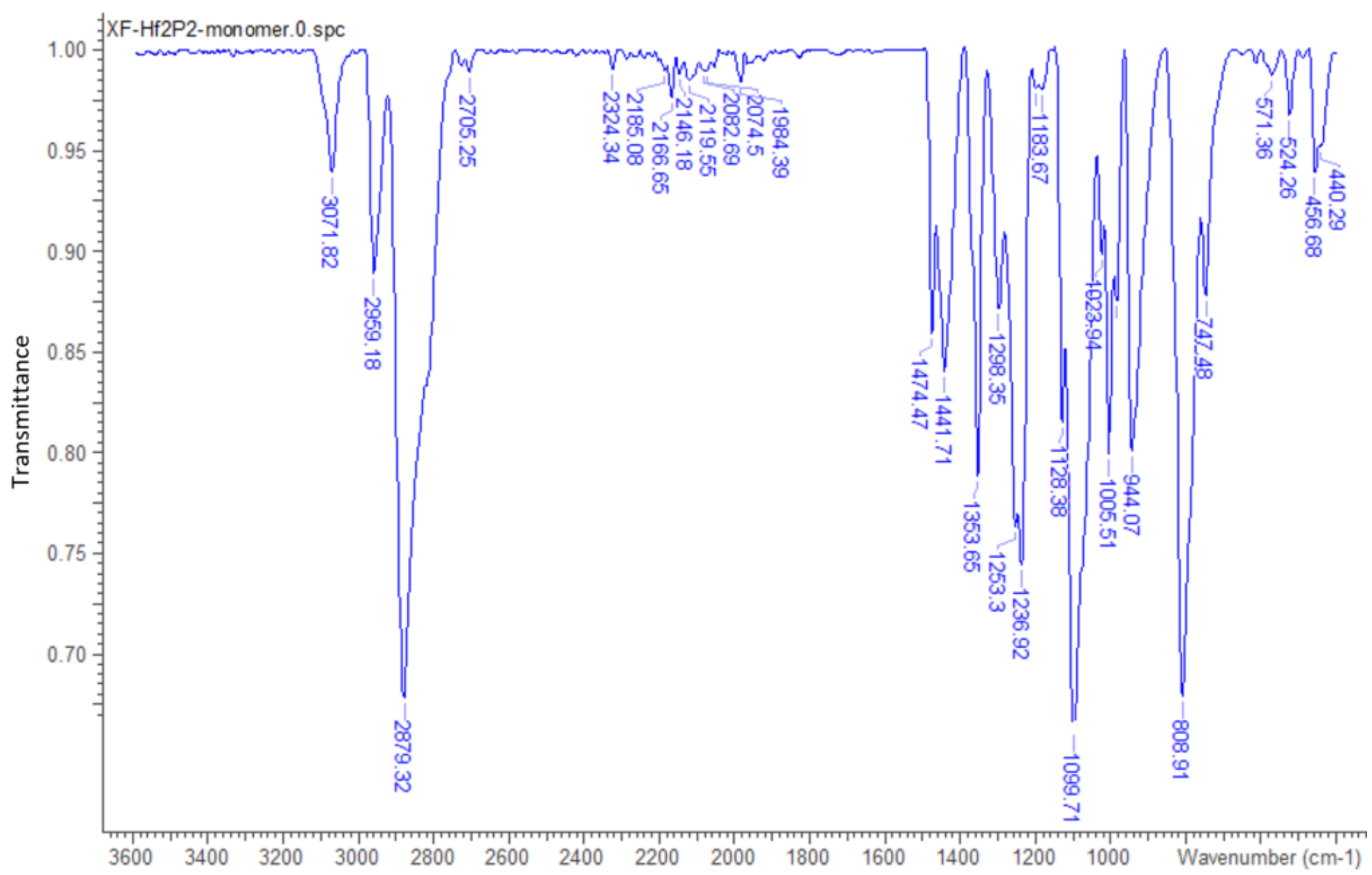


**Figure S13.** IR spectrum of complex **2-Hf**.

## IV. UV-Vis spectra

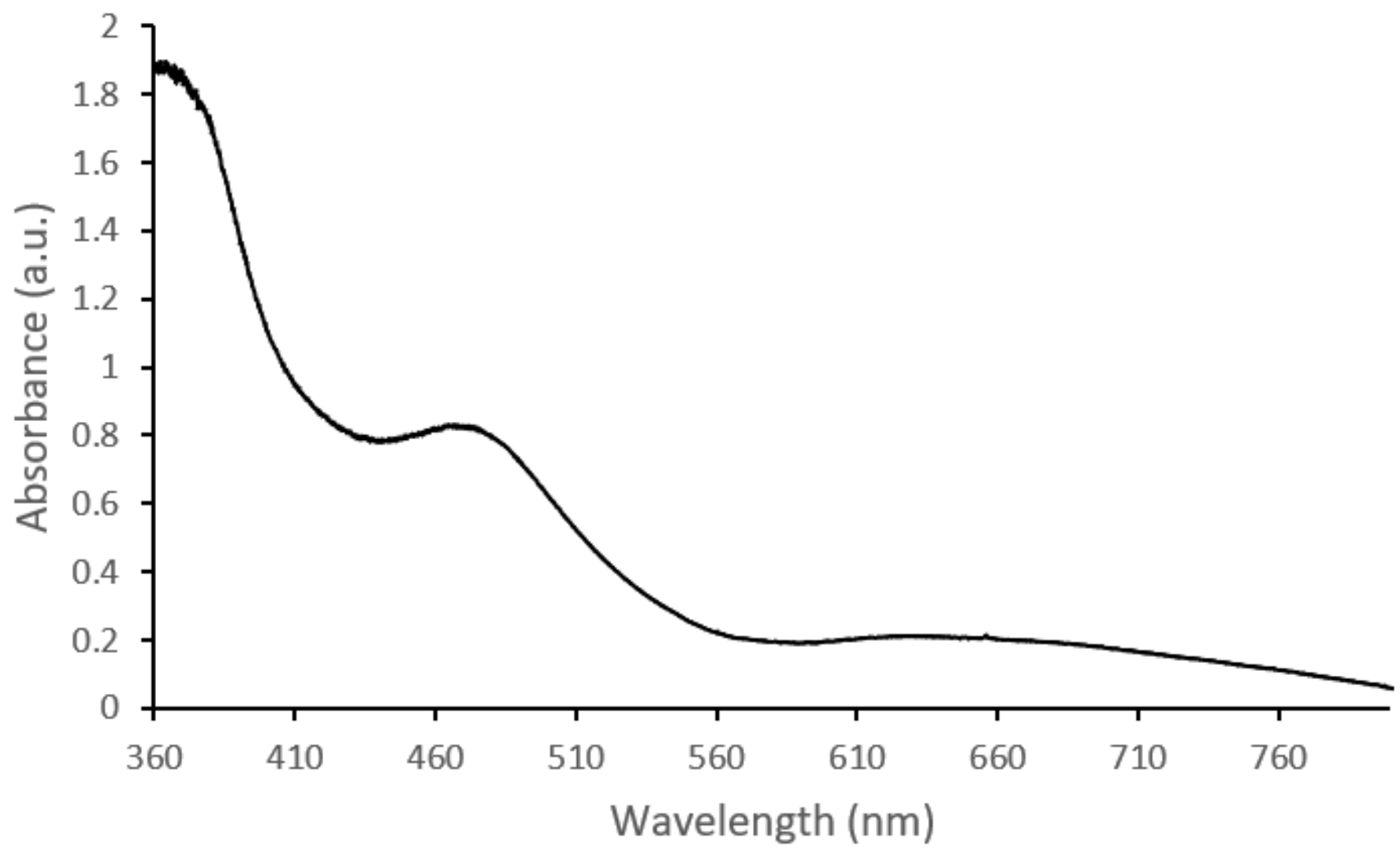


**Figure S14.** UV/vis absorption spectrum of **1-Ti** in the range 360-760 nm recorded at a concentration of 0.01 mg/mL in THF.

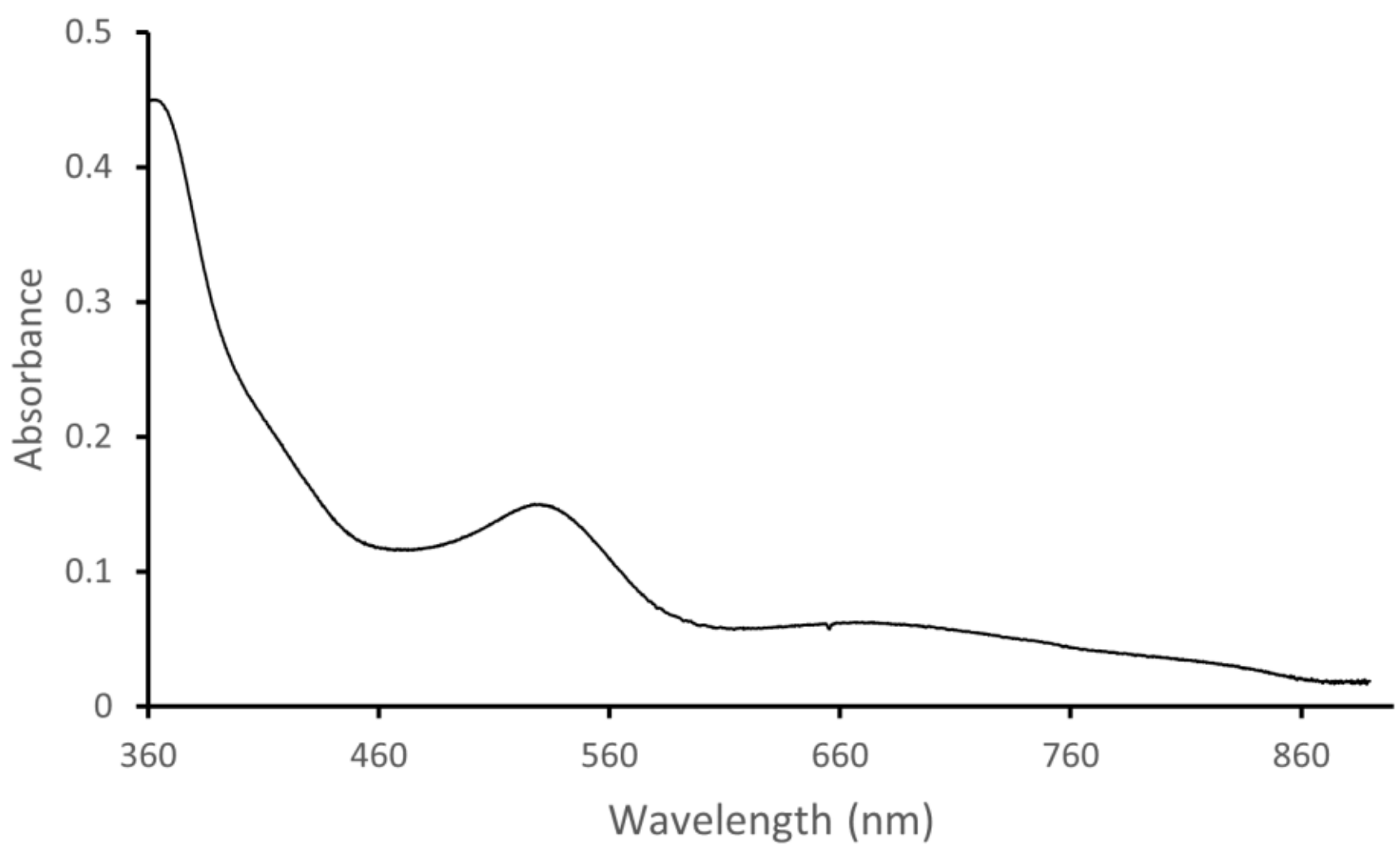


**Figure S15.** UV/vis absorption spectrum of **1-Zr** in the range 360-860 nm recorded at a concentration of 0.01 mg/mL in THF.

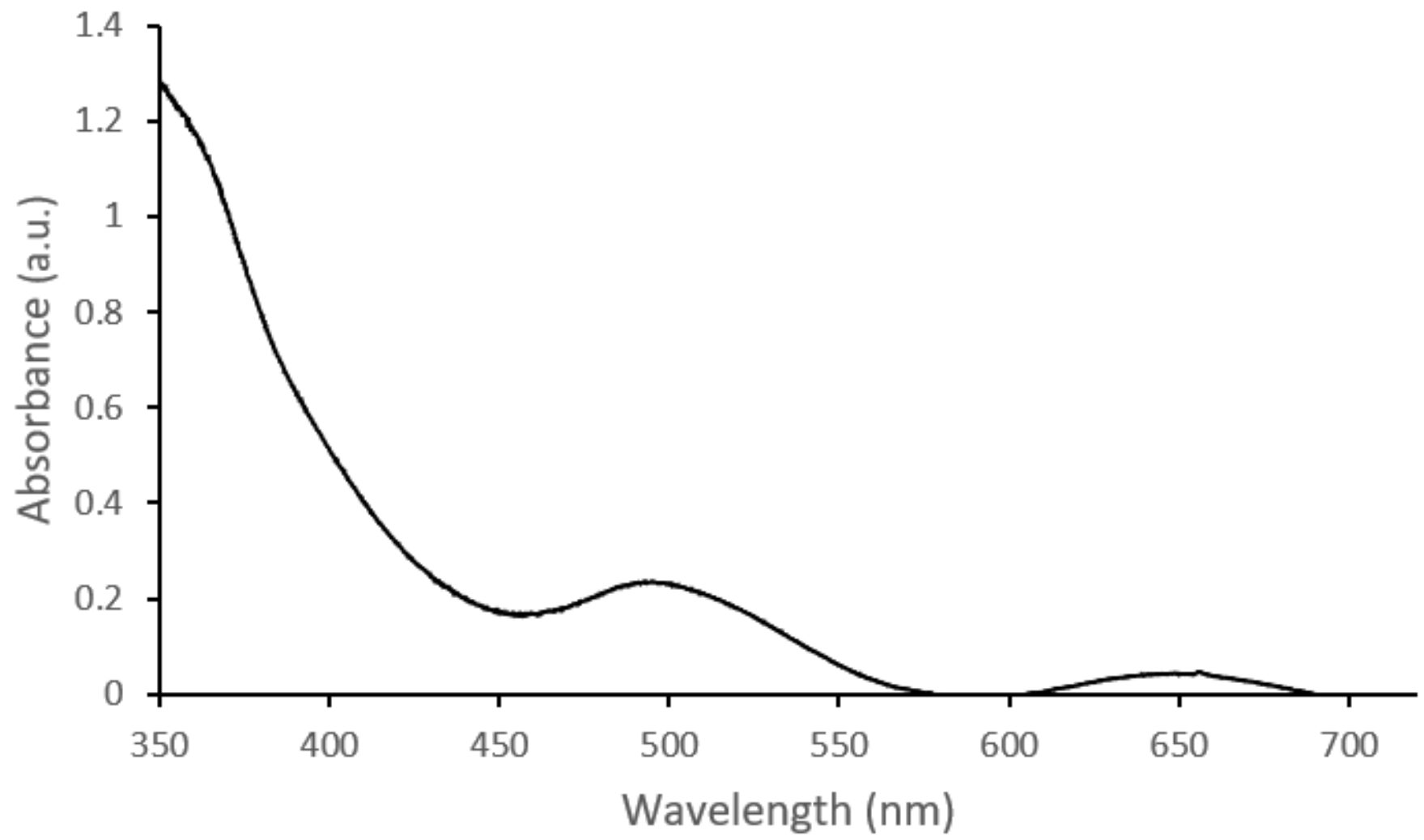


**Figure S16.** UV/vis absorption spectrum of **1-Hf** in the range 350-700 nm recorded at a concentration of 0.01 mg/mL in THF.

## V. EPR spectra

| parameter | value |
|---|---|
| g | 2.047 |
| $A_{iso}$(P) [MHz] | 68.1 |
| $A_{iso}$(M) [MHz] | 11.4 |
| lw [MHz] | 0.27 |

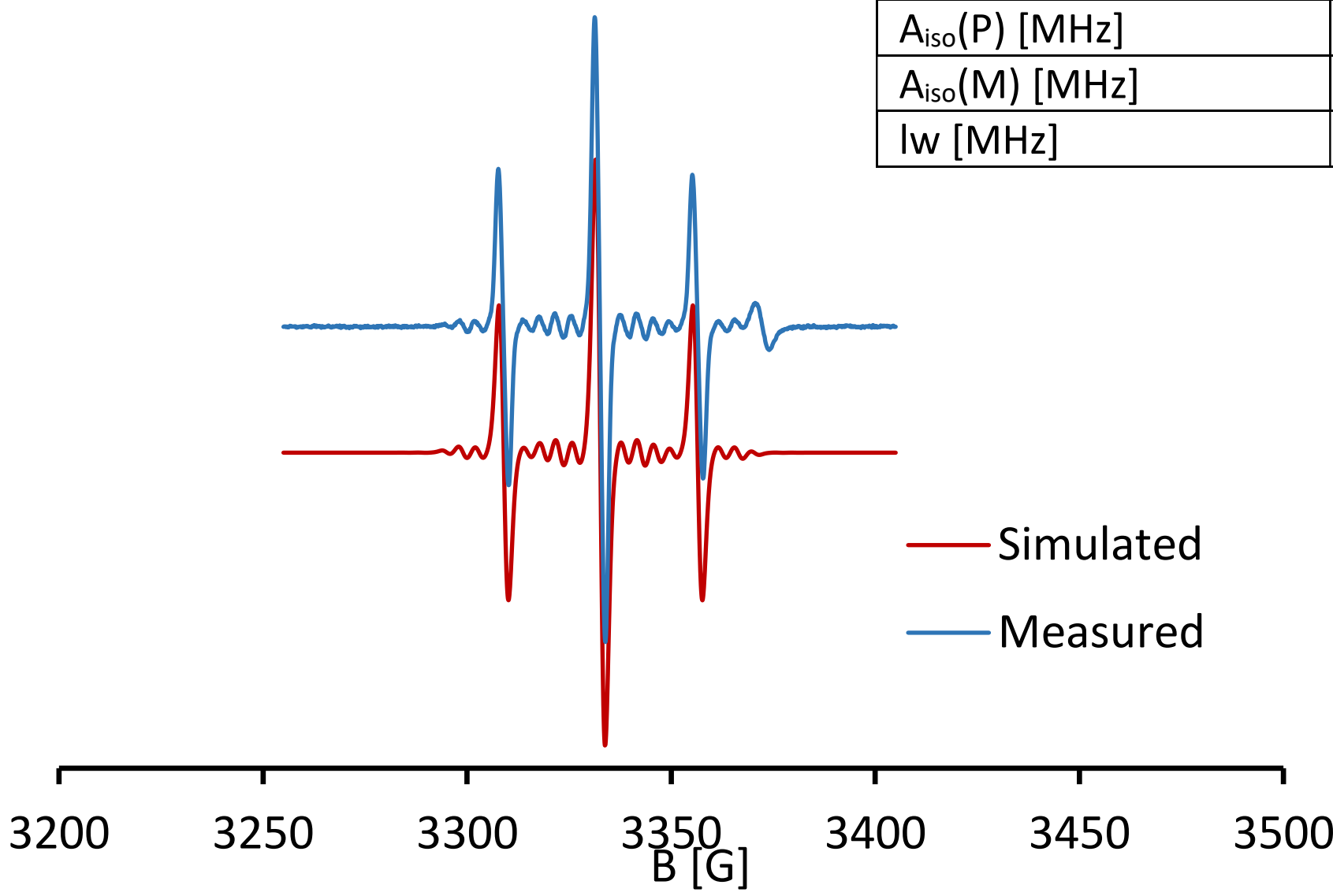


**Figure S17.** EPR spectrum of **1-Ti** in THF solution at ambient temperature (measured top, simulated bottom).

| parameter | value |
|---|---|
| g | 2.014 |
| $A_{iso}$(P) [MHz] | 66.4 |
| $A_{iso}$(M) [MHz] | 25.3 |
| lw [MHz] | 0.58 |

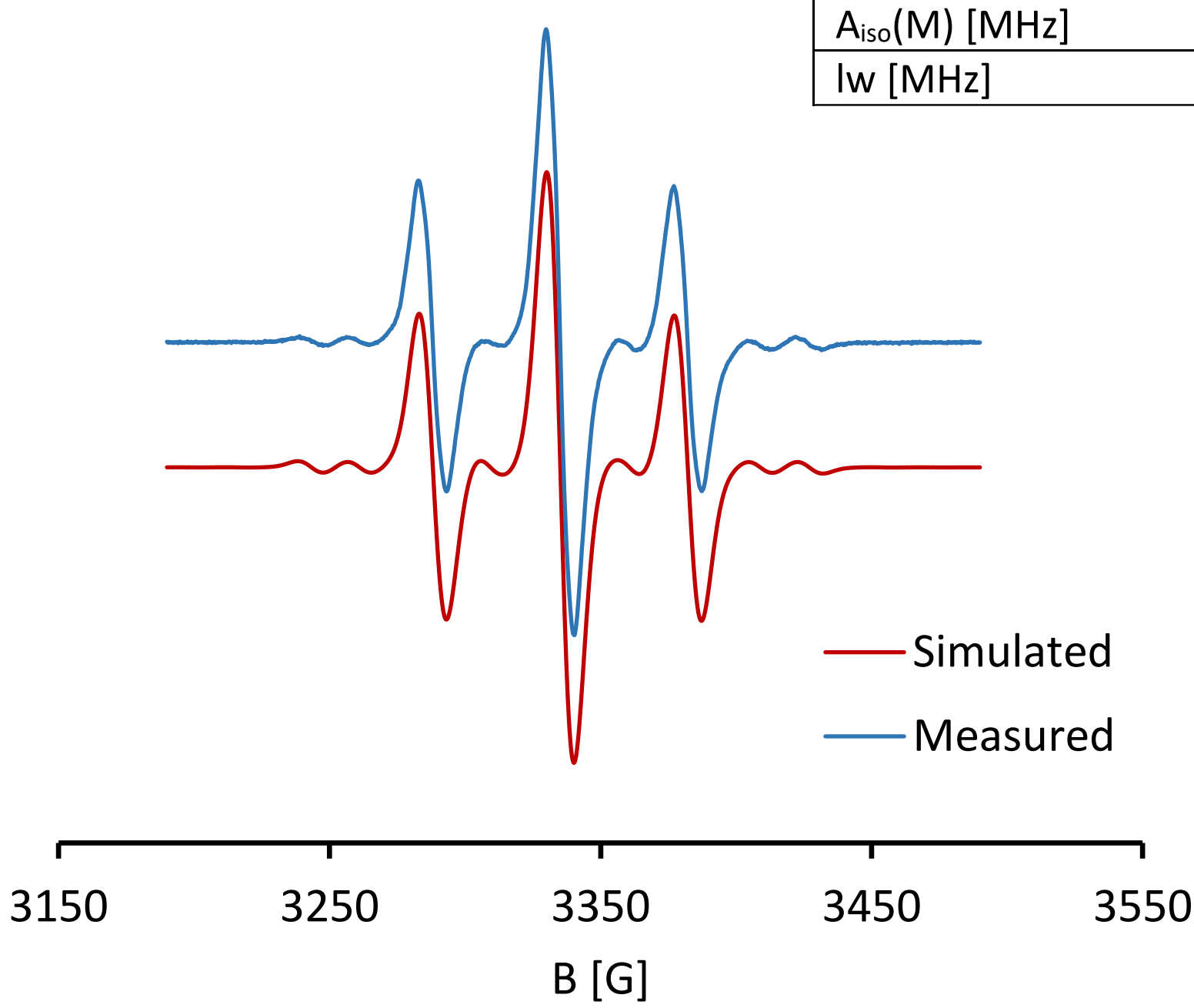


**Figure S18.** EPR spectrum of **1-Zr** in THF solution at ambient temperature (measured top, simulated bottom).

| parameter | value |
|---|---|
| g | 2.039 |
| $A_{iso}$(P) [MHz] | 62.3 |
| $A_{iso}$(M) [MHz] | 33.2 |
| lw [MHz] | 2.20 |

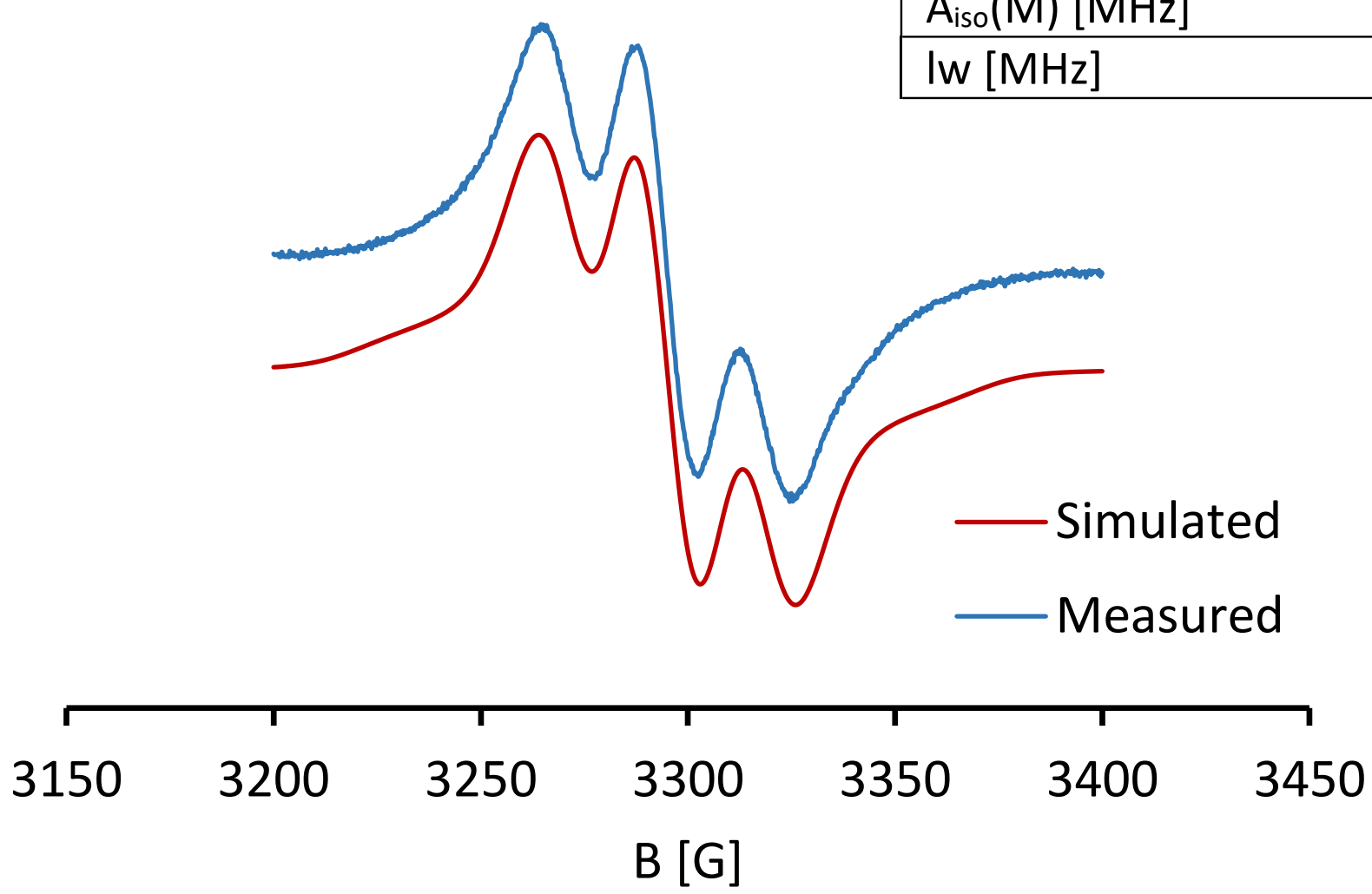


**Figure S19.** EPR spectrum of **1-Hf** in THF solution at ambient temperature (measured top, simulated bottom).

| parameter | value |
|---|---|
| g | 2.0024 |
| $A_{iso}$(P) [MHz] | 67.1649 |
| $A_{iso}$(M) [MHz] | 11 |
| lw [MHz] | 0.2379 |

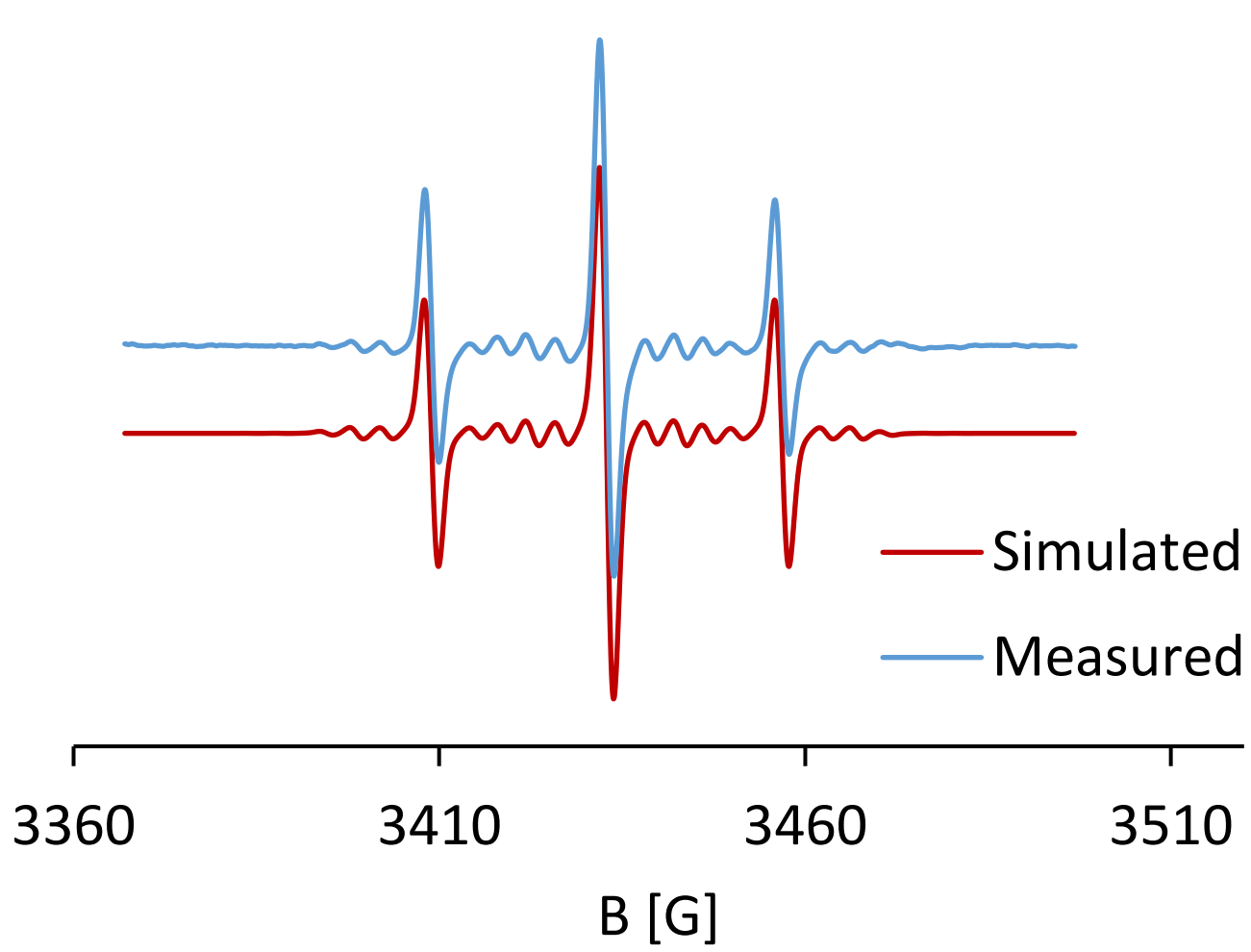


**Figure S20.** EPR spectrum of **2-Ti** in THF solution at ambient temperature (measured top, simulated bottom).

| parameter | value |
|---|---|
| g | 2.013779 |
| Aiso(P) [MHz] | 66.36353 |
| Aiso(M) [MHz] | 25.6856 |
| lw [MHz] | 0.697535 |

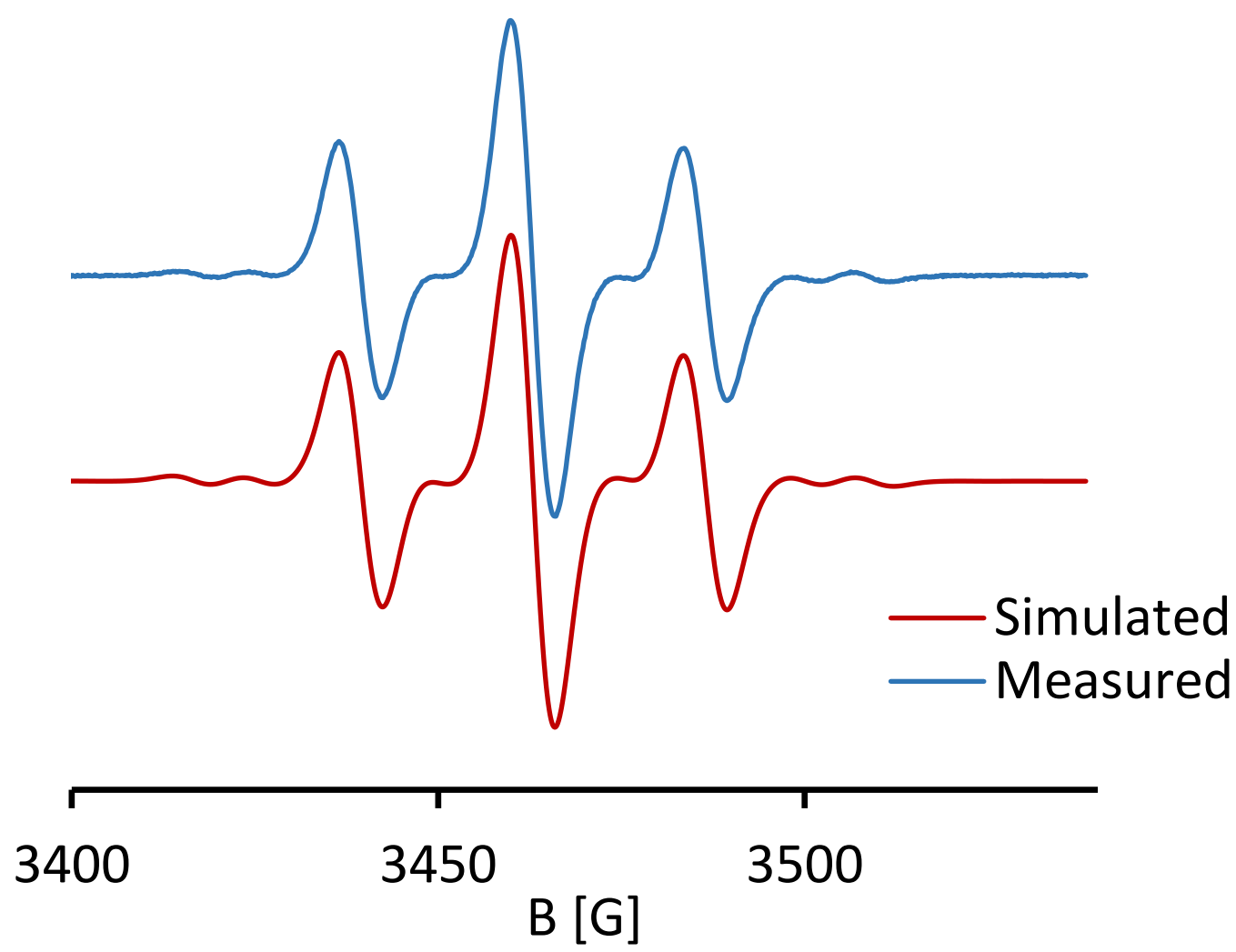


**Figure S21.** EPR spectrum of **2-Zr** in THF solution at ambient temperature (measured top, simulated bottom).

| parameter | value |
|---|---|
| g | 2.0423 |
| Aiso(P) [MHz] | 62.1915 |
| Aiso(M) [MHz] | 32.6714 |
| lw [MHz] | 2.2542 |

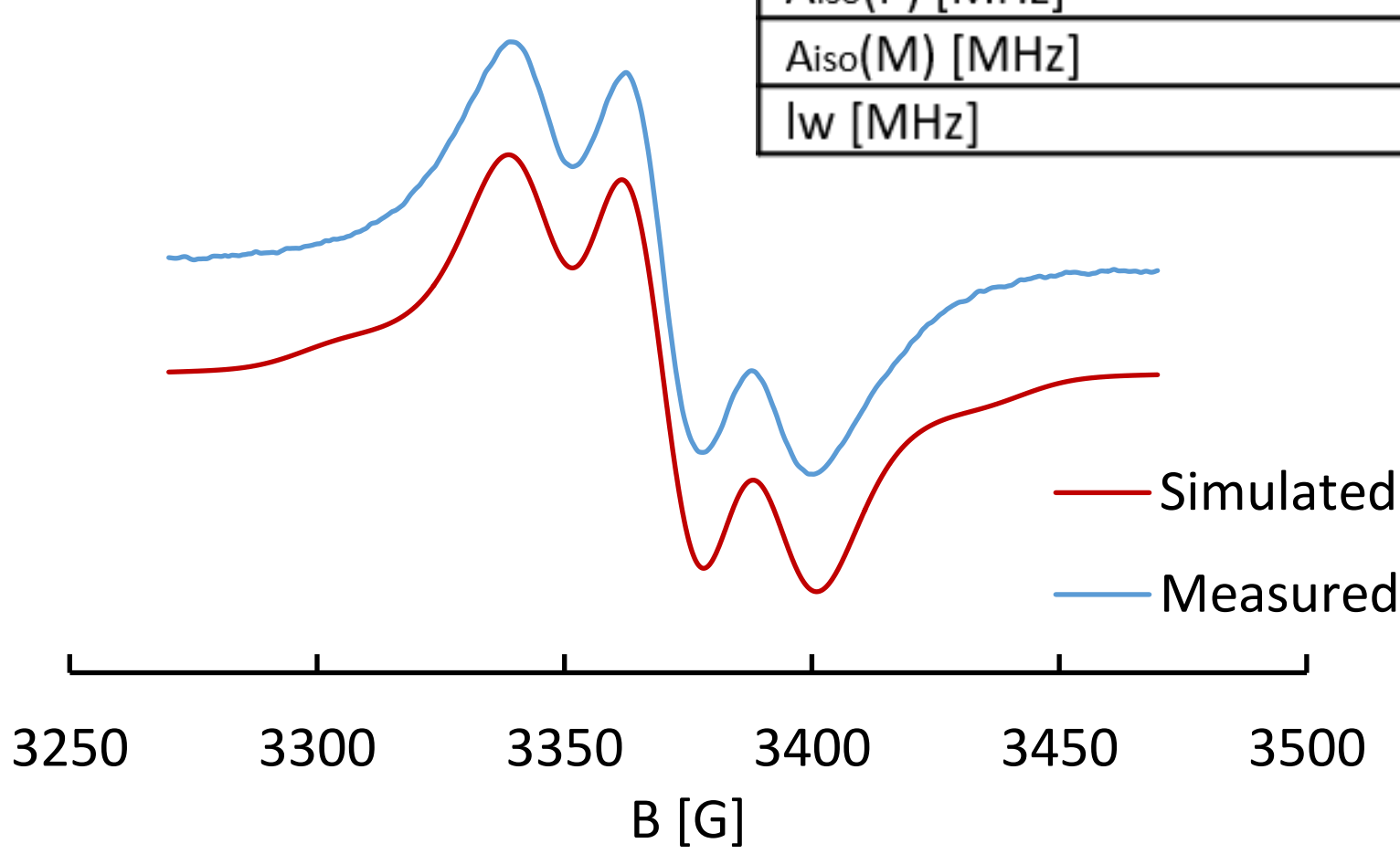


**Figure S22.** EPR spectrum of **2-Hf** in THF solution at ambient temperature (measured top, simulated bottom).

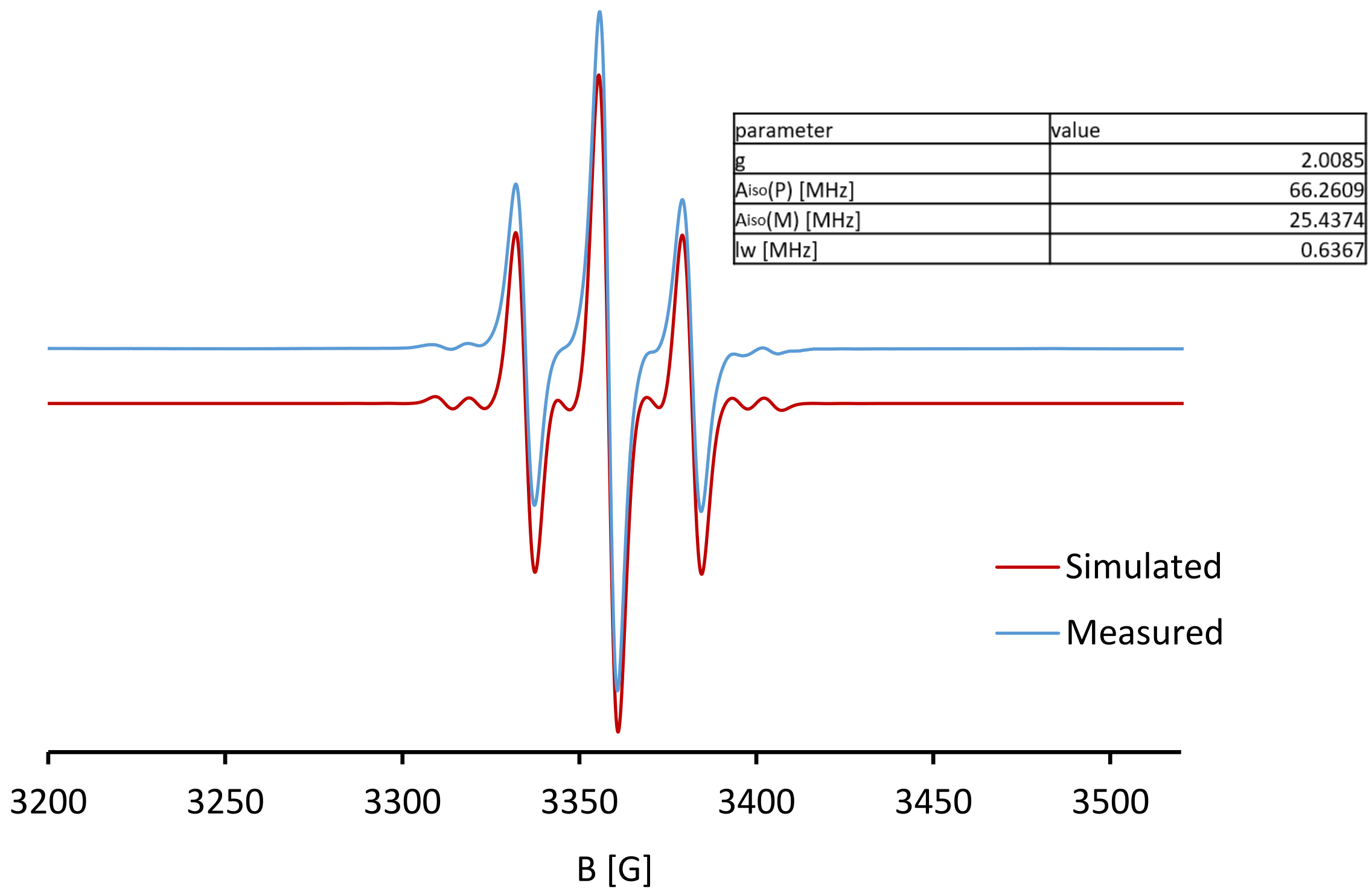


| parameter | value |
|---|---|
| g | 2.0085 |
| $A_{iso}$(P) [MHz] | 66.2609 |
| $A_{iso}$(M) [MHz] | 25.4374 |
| lw [MHz] | 0.6367 |

**Figure *S*23.** EPR spectrum of **1-Zr'** in THF solution at ambient temperature (measured top, simulated bottom).

## VI. NMR spectra

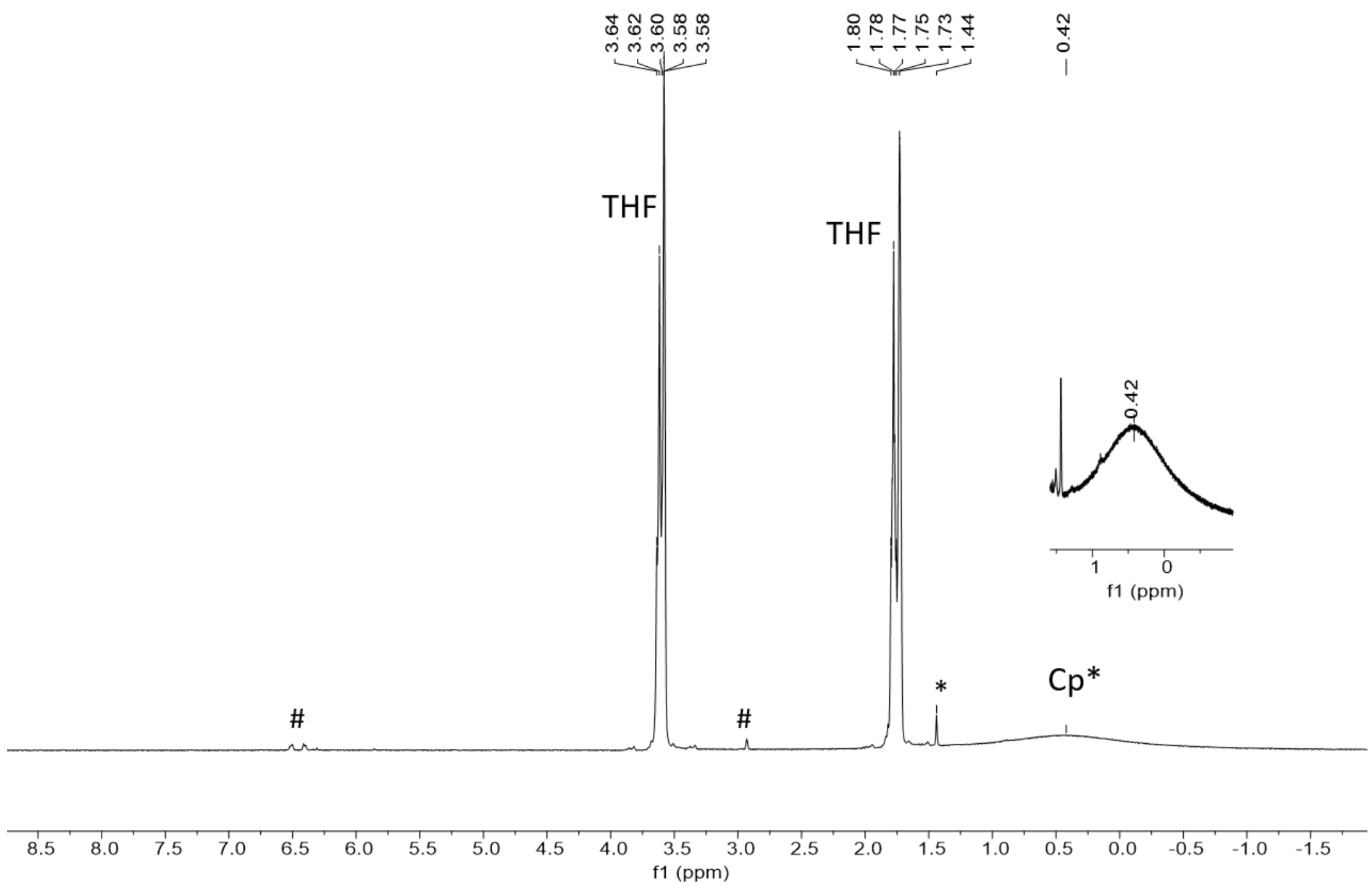


**Figure S24** $^{1}H$ NMR spectrum of **1-Zr** recorded in THF-$d_8$ at room temperature. Minor impurities of [Cp*Fe($\eta^5$-$P_5$)] are marked with * Minor impurities of CpH are marked with #.

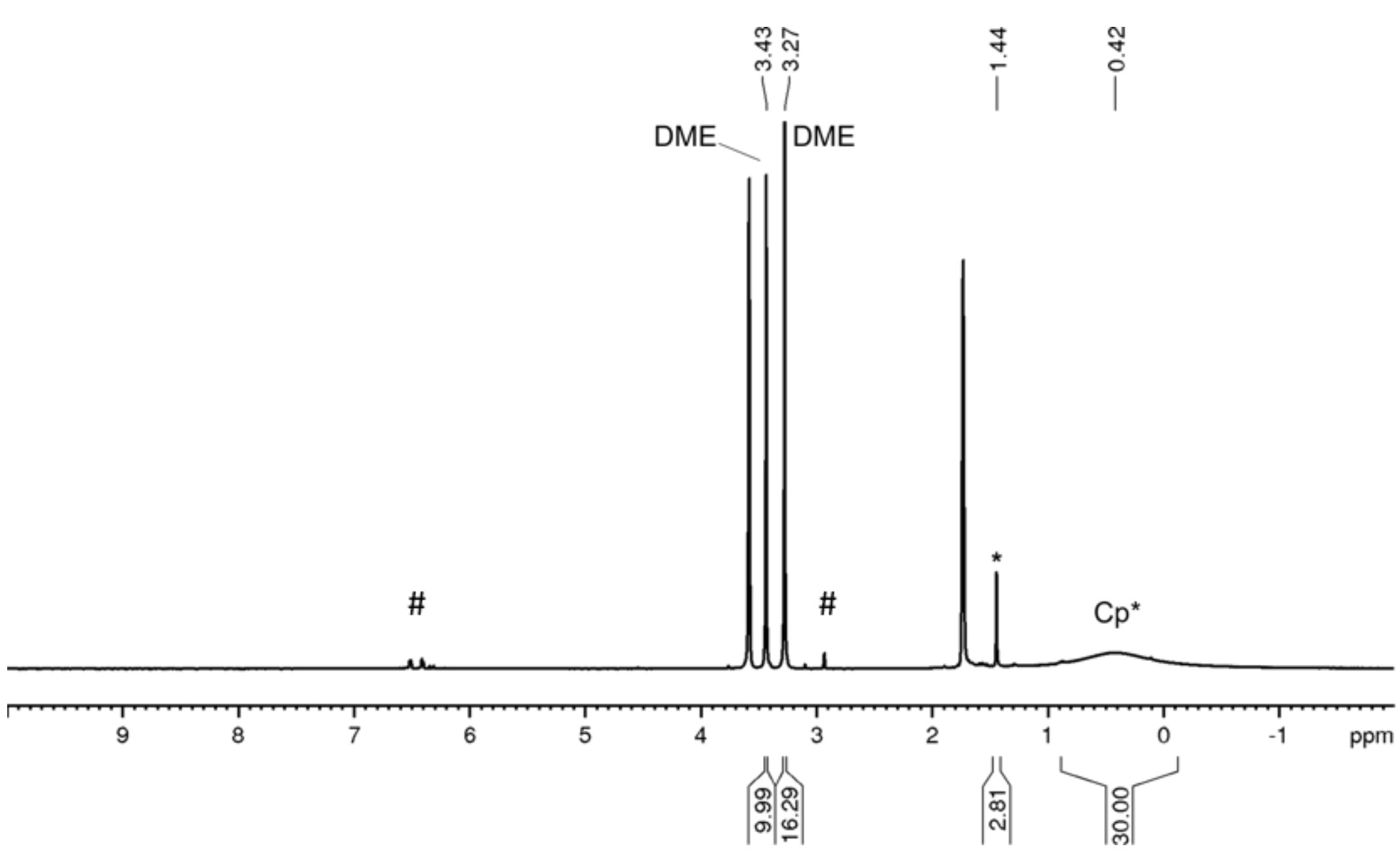


**Figure S25** $^{1}H$ NMR spectrum of **1-Zr'** recorded in THF-$d_8$ at room temperature. Minor impurities of [Cp*Fe($\eta^5$-$P_5$)] are marked with *. Minor impurities of CpH are marked with #.

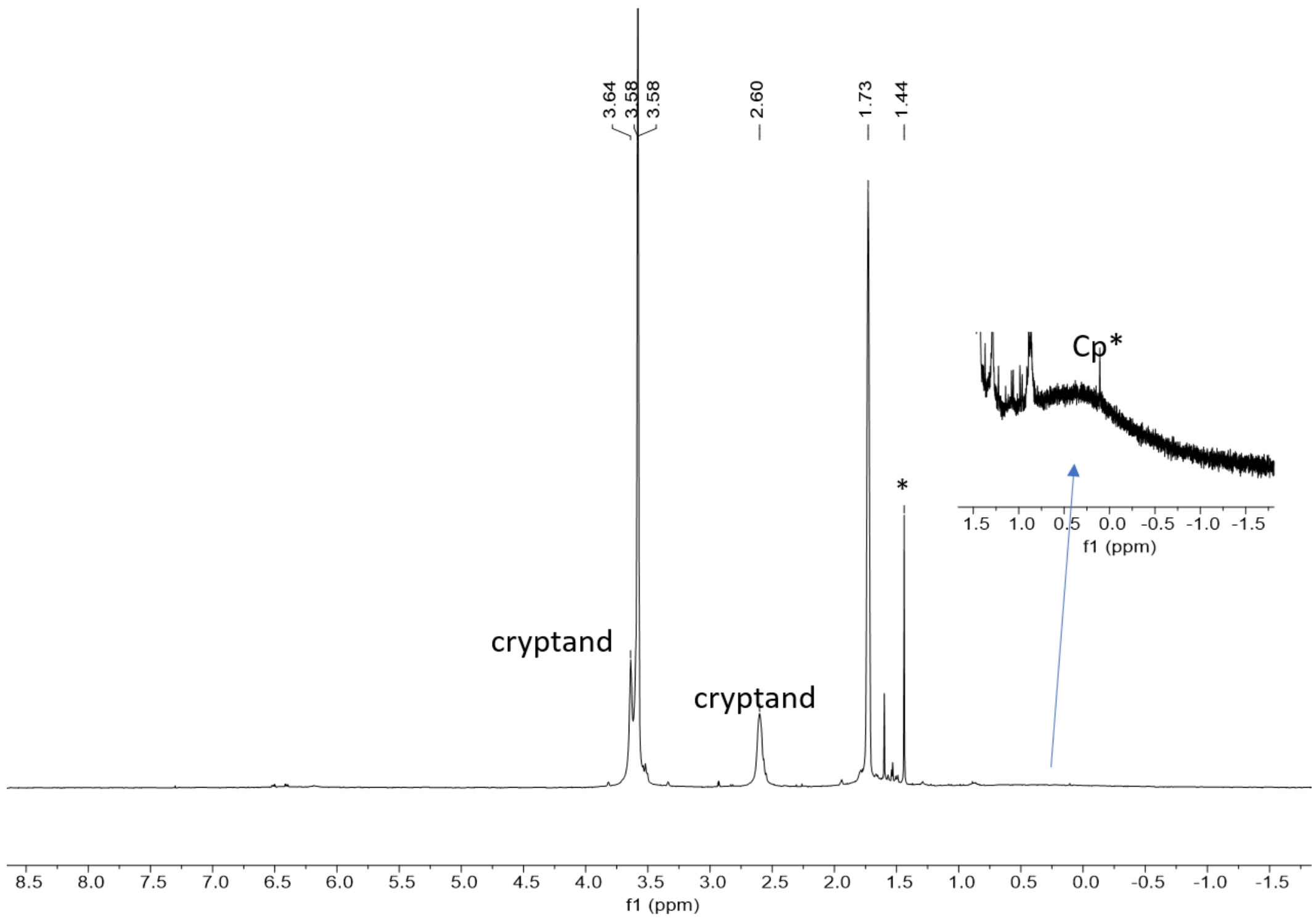


**Figure S26** $^{1}H$ NMR spectrum of **2-Zr** recorded in THF-$d_8$ at room temperature. Minor impurities of [Cp*Fe($\eta^5$-$P_5$)] are marked with *.

## VII. X-ray absorptions spectroscopy

Zr K X-ray Absorption Near Edge Structure (XANES) Spectra of the sample 1-Zr and a $CP_2ZrCL_2$ reference have been measured at the SUL-X beamline of the KIT synchrotron radiation facility at a 27 pole wiggler. The beam is monochromatized by a Si(111) double crystal monochromator (DCM) and was focused to about 0.25 mm (horizontal) and about 0.1 mm (vertical) to match with the capillary size using a KB mirror system at reduced slit openings and collimated for the measurements on the pellet to about 0.8 mm x 0.8 mm. Zr K XANES spectra were recorded in transmission using ionization chambers and in fluorescence mode at the Zr Kα fluorescence emission energies using a 7 element Silicon drift detector (Sirius, RaySpec). Because of significant self absorption in fluorescence mode, transmission data have been used for further processing. The energy was calibrated to the $1^{st}$ maximum of the $1^{st}$ derivative of Zr metall foil at 17.998 keV.

Zr K XANES spectra have been measured with energy steps of 0.3 eV across the edge and up to k 16 $Å^{-1}$ above the edge into the EXAFS region.

Two spectra were merged for the $CP_2ZrCL_2$ reference and 12 scans for the sample 1-Zr to get good statistics.

Data processing (energy calibration, pre- and post-edge background correction, normalization to edge jump 1) have been done with the ATHENA program of the free IFFEFIT software package.[6]

Data are supplied in ACSII format in the file ZrKXANES-data-final.txt.

## VIII. Quantum Chemical Calculations

General details

All computations were performed by Gaussian16 software package at PBE0/Def2-SVP level of theory with Grimme's dispersion correction in the gas phase.[7-10] The structures of **1-Ti**, **1-Zr** and **1-Hf** were optimised and checked to not possess imaginary vibrational frequencies. Natural Population Analysis (NPA) was done with NBO7.0[11] and spin density calculation were carried out with MultiWFN.[12, 13]

**Table S3.** Spin density distributions, contributions >0.01e.

| | Ti | Zr | Hf |
|---|---|---|---|
| ρ(Ti) | +0.58 | +0.44 | +0.42 |
| ρ(P) | –0.06 | +0.02 | +0.02 |

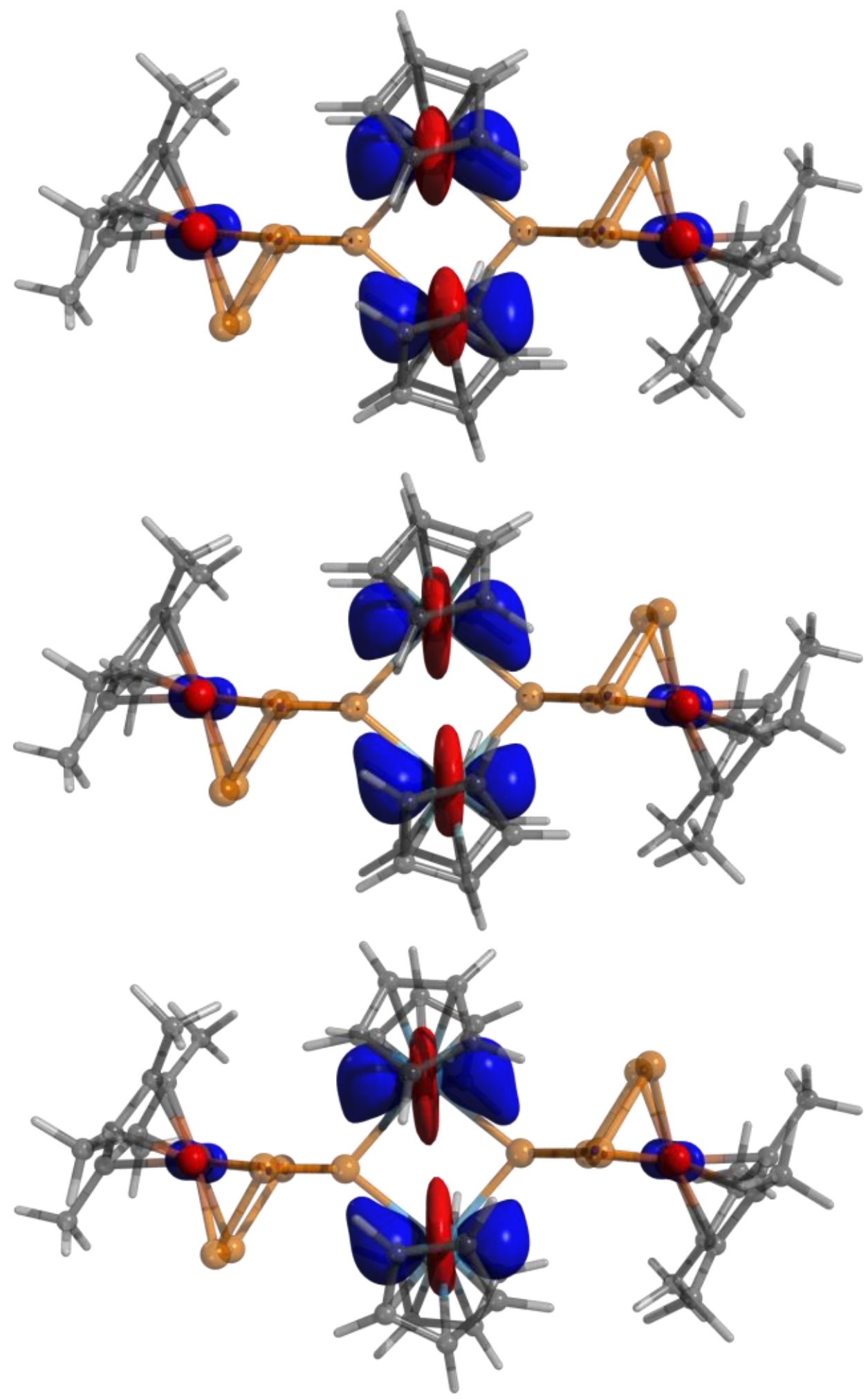

**Figure S27**. SOMO of **1-Ti** (top), **1-Zr** (middle), **1-Hf** (bottom) radical anions (isovalue 0.05).

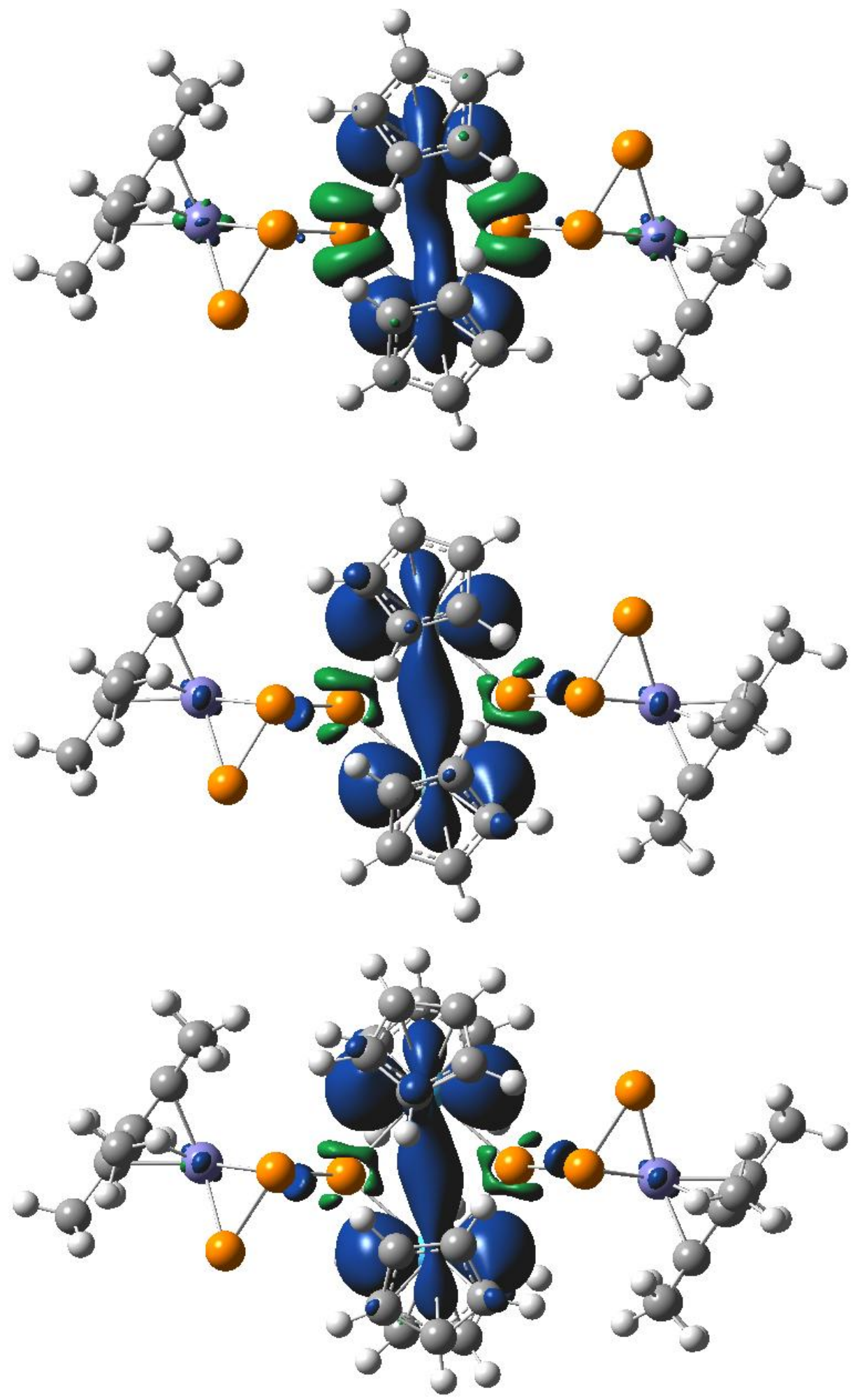

**Figure S28.** Spin density distribution of **1-Ti** (top), **1-Zr** (middle), **1-Hf** (bottom) radical anions (isovalue 0.0012).

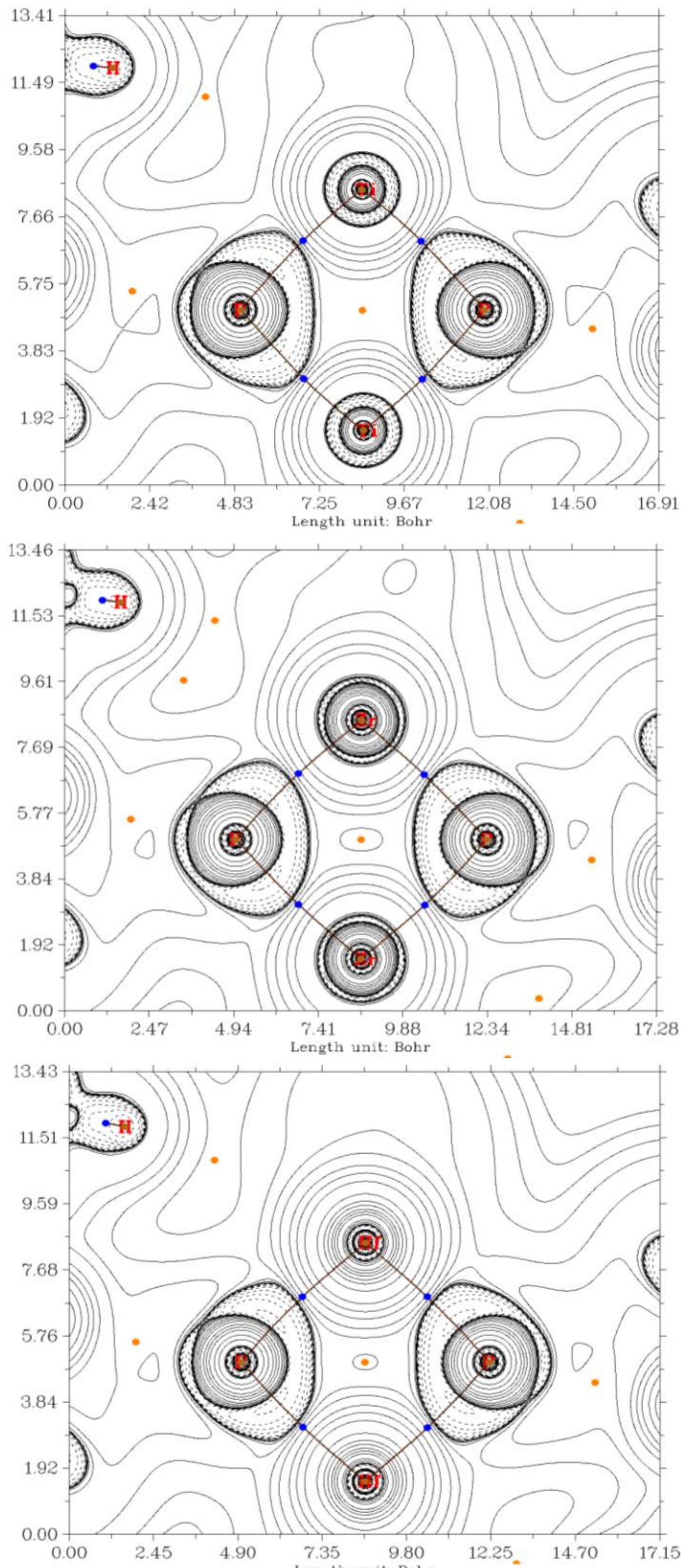


**Figure S29**. Plot of ∇(ρ) of the $M_2P_2$ core including critical points, **1-Ti** (top), **1-Zr** (middle), **1-Hf** (bottom), Def2-TZVP basis set; orange: (3,+1) critical point, blue: (3,–1) critical point.

**Table S4.** Properties of critical point in centre of $M_2P_2$ plane.

| Def2-SVP | **Ti** | **Zr** | **Hf** |
|---|---|---|---|
| $\rho$ | 0.2083548279E-01 | 0.2087429438E-01 | 0.2171048848E-01 |
| $\rho(\alpha)$ | 0.1148994633E-01 | 0.1247974244E-01 | 0.1299781162E-01 |
| $\rho(\beta)$ | 0.9345536460E-02 | 0.8394551942E-02 | 0.8712676859E-02 |
| $\nabla(\rho)$ | 0.1912896396E-01 | 0.1520628669E-01 | 0.1506336677E-01 |
| ELF | 0.2968415398E+00 | 0.3459436652E+00 | 0.3649582351E+00 |

| Def2-TZVP | **Ti** | **Zr** | **Hf** |
|---|---|---|---|
| $\rho$ | 0.1971730052E-01 | 0.2039703352E-01 | 0.2243647238E-01 |
| $\rho(\alpha)$ | 0.1101943438E-01 | 0.1246674148E-01 | 0.1365150547E-01 |
| $\rho(\beta)$ | 0.8697866134E-02 | 0.7930292043E-02 | 0.8784966911E-02 |
| $\nabla(\rho)$ | 0.2187625375E-01 | 0.1661792043E-01 | 0.1872147188E-01 |
| ELF | 0.2714538876E+00 | 0.3390582753E+00 | 0.3564221295E+00 |

**Table S5.** AIM data on Ti, Zr, Hf compounds, Def2-TZVP basis set.

| | **$Cp_2TiCl_2$** | **$[(Cp_2TiCl)_2]$** | **$[(Cp_2TiCl)_2]^{-}$** | **$[(Cp_2TiCl)_2]^{2-}$** | **$[(Cp_2TiP_5)_2]$** | **$[(Cp_2TiP_5)_2]^{-}$** | **$[(Cp_2TiP_5)_2]^{2-}$** |
|---|---|---|---|---|---|---|---|
| ox.state | **+4** | **+3** | **+2.5** | **+2** | **+3** | **+2.5** | **+2** |
| q in e | 1.869976 | 1.659777<br>1.659713 | 1.591825<br>1.591699 | 1.521745<br>1.521934 | 1.673942<br>1.673930 | 1.603976<br>1.603863 | 1.533676<br>1.533638 |

| | **$Cp_2ZrCl_2$** | **$[(Cp_2ZrCl)_2]$** | **$[(Cp_2ZrCl)_2]^{-}$** | **$[(Cp_2ZrCl)_2]^{2-}$** | **$[(Cp_2ZrP_5)_2]$** | **$[(Cp_2ZrP_5)_2]^{-}$** | **$[(Cp_2ZrP_5)_2]^{2-}$** |
|---|---|---|---|---|---|---|---|
| ox.state | **+4** | **+3** | **+2.5** | **+2** | **+3** | **+2.5** | **+2** |
| q in e | 2.180742 | 1.825158<br>1.825255 | 1.793978<br>1.793465 | 1.740036<br>1.740017 | 1.940752<br>1.940596 | 1.829097<br>1.829357 | 1.729600<br>1.729828 |

| | **$Cp_2HfCl_2$** | **$[(Cp_2HfCl)_2]$** | **$[(Cp_2HfCl)_2]^{-}$** | **$[(Cp_2HfCl)_2]^{2-}$** | **$[(Cp_2HfP_5)_2]$** | **$[(Cp_2HfP_5)_2]^{-}$** | **$[(Cp_2HfP_5)_2]^{2-}$** |
|---|---|---|---|---|---|---|---|
| ox.state | **+4** | **+3** | **+2.5** | **+2** | **+3** | **+2.5** | **+2** |
| q in e | 2.277086 | 1.909445<br>1.909809 | 1.895645<br>1.895492 | 1.852664<br>1.852604 | 2.040403<br>2.040085 | 1.916055<br>1.916049 | 1.820595<br>1.820270 |

Cartesian coordinates:

$[(Cp_2Ti)_2(P5)_2(FeCp^*)_2]^{\cdot -}$

-1 2

| | | | |
|---|---|---|---|
| Fe | 5.11080400 | 0.16394300 | -0.00013800 |
| C | 6.71208300 | -0.37100500 | -1.15965700 |
| C | 7.13328200 | 0.35977100 | 0.00059400 |
| P | 3.41408400 | 0.22690500 | 1.51865300 |
| C | 6.71126300 | -0.37105400 | 1.16050000 |
| P | 4.40721900 | 2.09339500 | 1.08412400 |
| C | 6.02346200 | -1.54630000 | 0.71491600 |
| P | 4.40722900 | 2.09329800 | -1.08456500 |
| C | 6.02395300 | -1.54626600 | -0.71461800 |
| P | 3.41403200 | 0.22672800 | -1.51886400 |
| Ti | 0.22883500 | -1.77783700 | -0.00019900 |
| C | -0.66492100 | -1.98199900 | -2.19294400 |
| C | -0.65306300 | -3.26364000 | -1.59940300 |
| C | 0.70376100 | -3.63204200 | 1.40680700 |
| C | -0.65240700 | -3.26419500 | 1.59897800 |
| C | -0.66471000 | -1.98260800 | 2.19260100 |
| C | 6.95057900 | 0.00264500 | -2.58410000 |
| P | 1.80756300 | 0.19853100 | -0.00007200 |
| C | 7.89987100 | 1.63886000 | 0.00073800 |
| C | 6.94864100 | 0.00239700 | 2.58518700 |
| C | 5.47570300 | -2.61620900 | 1.59810500 |
| C | 5.47649800 | -2.61613500 | -1.59804700 |
| H | 6.08456600 | -0.25868500 | -3.21098900 |
| H | 7.83492200 | -0.51424000 | -2.99634600 |
| H | 7.10721700 | 1.08536200 | -2.68824700 |
| H | 7.65208500 | 2.24531700 | -0.88224500 |

| | | | |
|---|---|---|---|
| H | 8.98890100 | 1.45822900 | 0.00057600 |
| H | 7.65228700 | 2.24506500 | 0.88394800 |
| H | 7.10791300 | 1.08472400 | 2.68928100 |
| H | 7.83107100 | -0.51664500 | 2.99881600 |
| H | 6.08106600 | -0.25657200 | 3.21092500 |
| H | 6.26756300 | -3.32133600 | 1.90636400 |
| H | 4.69656500 | -3.19802300 | 1.08558700 |
| H | 5.02612500 | -2.19203700 | 2.50757700 |
| H | 4.69672600 | -3.19750400 | -1.08600200 |
| H | 6.26829800 | -3.32165700 | -1.90557100 |
| H | 5.02775600 | -2.19198700 | -2.50794500 |
| H | -1.56649500 | -1.46085600 | -2.51416400 |
| C | 0.66938100 | -1.53891000 | -2.30983700 |
| H | -1.53757900 | -3.85392600 | -1.36491800 |
| C | 0.70296900 | -3.63200300 | -1.40735700 |
| H | 1.05660100 | -4.58518600 | -1.02627300 |
| C | 1.52097100 | -2.55947600 | -1.81428300 |
| H | 2.60723400 | -2.50846100 | -1.77344000 |
| H | 1.01492400 | -0.60147400 | -2.73593300 |
| H | 1.05775900 | -4.58501200 | 1.02552700 |
| C | 1.52139200 | -2.55927000 | 1.81379800 |
| H | -1.53668400 | -3.85479900 | 1.36438900 |
| H | -1.56647700 | -1.46174100 | 2.51371300 |
| C | 0.66946100 | -1.53904700 | 2.30945800 |
| H | 1.01456200 | -0.60147400 | 2.73561300 |
| H | 2.60763000 | -2.50789900 | 1.77278700 |
| P | -1.80761800 | -0.19842900 | -0.00010300 |
| Ti | -0.22890400 | 1.77787500 | -0.00004600 |
| P | -3.41422000 | -0.22671500 | -1.51871100 |

| | | | |
|---|---|---|---|
| P | -3.41388400 | -0.22695400 | 1.51887600 |
| C | 0.66505300 | 1.98263800 | 2.19252400 |
| C | 0.65234600 | 3.26424900 | 1.59894400 |
| C | -0.70394400 | 3.63183400 | 1.40709400 |
| C | -1.52124800 | 2.55888500 | 1.81424300 |
| C | -0.66899500 | 1.53880500 | 2.30965200 |
| C | -0.70341600 | 3.63215300 | -1.40708800 |
| C | 0.65272000 | 3.26414300 | -1.59912000 |
| C | 0.66492500 | 1.98256700 | -2.19276700 |
| C | -0.66928000 | 1.53915600 | -2.30975100 |
| C | -1.52114000 | 2.55947500 | -1.81416700 |
| Fe | -5.11078700 | -0.16399000 | 0.00028900 |
| P | -4.40729100 | -2.09327100 | -1.08431700 |
| P | -4.40704100 | -2.09349400 | 1.08436900 |
| H | 1.56695700 | 1.46196600 | 2.51359100 |
| H | 1.53646700 | 3.85506500 | 1.36431100 |
| H | -1.05822800 | 4.58477900 | 1.02602000 |
| H | -2.60748900 | 2.50727200 | 1.77362500 |
| H | -1.01394800 | 0.60118500 | 2.73581100 |
| H | -1.05731000 | 4.58517200 | -1.02583300 |
| H | 1.53704900 | 3.85463000 | -1.36443800 |
| H | 1.56665300 | 1.46163300 | -2.51386900 |
| H | -1.01442700 | 0.60163200 | -2.73596700 |
| H | -2.60738900 | 2.50823300 | -1.77326600 |
| C | -6.71198100 | 0.37078500 | 1.16000800 |
| C | -7.13326100 | -0.35984400 | -0.00030500 |
| C | -6.71134100 | 0.37113900 | -1.16014900 |
| C | -6.02351100 | 1.54633200 | -0.71446400 |
| C | -6.02389800 | 1.54611100 | 0.71506800 |

| | | | |
|---|---|---|---|
| C | -6.95033200 | -0.00303300 | 2.58443200 |
| C | -7.89982900 | -1.63894600 | -0.00056000 |
| C | -6.94882200 | -0.00212700 | -2.58486800 |
| C | -5.47581600 | 2.61637300 | -1.59753100 |
| C | -5.47641400 | 2.61588200 | 1.59859700 |
| H | -6.08409000 | 0.25785100 | 3.21119500 |
| H | -7.83439200 | 0.51411700 | 2.99695100 |
| H | -7.10737000 | -1.08570600 | 2.68841800 |
| H | -7.65192900 | -2.24553700 | 0.88229900 |
| H | -8.98886200 | -1.45833700 | -0.00024900 |
| H | -7.65233100 | -2.24501100 | -0.88389100 |
| H | -7.10814900 | -1.08443400 | -2.68908600 |
| H | -7.83125300 | 0.51700700 | -2.99837900 |
| H | -6.08127200 | 0.25687900 | -3.21062600 |
| H | -6.26765100 | 3.32167800 | -1.90544600 |
| H | -4.69648000 | 3.19795800 | -1.08505200 |
| H | -5.02649800 | 2.19235300 | -2.50720100 |
| H | -4.69662100 | 3.19728100 | 1.08661800 |
| H | -6.26820000 | 3.32139700 | 1.90617800 |
| H | -5.02770500 | 2.19163900 | 2.50846600 |

$[(Cp_2Zr)_2(P5)_2(FeCp^*)_2]^{\cdot -}$

-1 2

| | | | |
|---|---|---|---|
| Fe | -5.17100700 | 0.17181400 | -0.00005100 |
| C | -6.76764700 | -0.37776700 | 1.16011900 |
| C | -7.19681600 | 0.34691600 | -0.00079900 |
| P | -3.48017300 | 0.24937200 | -1.53877600 |

| | | | |
|---|---|---|---|
| C | -6.76676600 | -0.37990700 | -1.16007200 |
| P | -4.48691200 | 2.11116700 | -1.08203600 |
| C | -6.06562700 | -1.54725900 | -0.71314100 |
| P | -4.48682900 | 2.11160800 | 1.08104100 |
| C | -6.06624700 | -1.54594500 | 0.71592100 |
| P | -3.48010600 | 0.25001200 | 1.53851600 |
| Zr | -0.22392200 | -1.81539200 | -0.00016300 |
| C | -0.69646100 | -3.71479900 | -1.52589600 |
| C | 0.66126400 | -3.35160100 | -1.73650200 |
| C | 0.67541300 | -2.07114300 | -2.33836000 |
| C | -0.66201600 | -1.62973500 | -2.46527900 |
| C | -1.51589400 | -2.64648700 | -1.96197700 |
| C | -0.69744700 | -3.71456600 | 1.52547700 |
| C | 0.66043800 | -3.35200300 | 1.73617400 |
| C | -0.66207400 | -1.62956600 | 2.46495100 |
| C | -1.51640000 | -2.64589000 | 1.96156100 |
| C | 0.67517100 | -2.07156900 | 2.33804300 |
| C | -7.01109500 | -0.00508000 | 2.58395300 |
| P | -1.91023900 | 0.21231900 | -0.00017100 |
| C | -7.97683200 | 1.61774000 | -0.00214100 |
| C | -7.00908100 | -0.00972500 | -2.58475100 |
| C | -5.50206400 | -2.61307600 | -1.59125200 |
| C | -5.50383200 | -2.61018200 | 1.59669000 |
| H | -6.14276400 | -0.25597900 | 3.21194700 |
| H | -7.18022700 | 1.07590800 | 2.68660800 |
| H | -7.73480100 | 2.22801000 | 0.87979800 |
| H | -9.06387200 | 1.42565500 | -0.00111600 |
| H | -7.73601600 | 2.22543800 | -0.88617200 |
| H | -7.17766600 | 1.07115600 | -2.68947200 |

| | | | |
|---|---|---|---|
| H | -7.88764000 | -0.53638500 | -2.99689900 |
| H | -6.14038700 | -0.26207300 | -3.21165800 |
| H | -6.27584400 | -3.35044400 | -1.86868300 |
| H | -4.69119300 | -3.15732000 | -1.08651500 |
| H | -5.08840600 | -2.18991000 | -2.51784500 |
| H | -4.69482000 | -3.15787800 | 1.09272000 |
| H | -6.27890900 | -3.34497000 | 1.87732200 |
| H | -5.08815300 | -2.18502300 | 2.52148400 |
| H | -1.04894500 | -4.65969500 | -1.11839800 |
| H | 1.54509200 | -3.94180700 | -1.49548700 |
| H | 1.57864700 | -1.53598900 | -2.63532500 |
| H | -1.00507900 | -0.68792900 | -2.88819800 |
| H | -2.60306800 | -2.59702200 | -1.93330500 |
| H | -1.05043100 | -4.65922100 | 1.11785700 |
| H | 1.54400500 | -3.94250500 | 1.49492200 |
| H | -1.00467900 | -0.68761100 | 2.88790000 |
| H | -2.60355500 | -2.59594100 | 1.93290200 |
| H | 1.57862500 | -1.53680500 | 2.63505300 |
| Zr | 0.22394400 | 1.81544900 | -0.00014600 |
| P | 1.91025600 | -0.21226600 | -0.00002100 |
| C | 0.69705000 | 3.71475900 | 1.52552400 |
| C | -0.66078700 | 3.35195800 | 1.73613600 |
| C | -0.67531300 | 2.07153000 | 2.33804600 |
| C | 0.66198800 | 1.62974000 | 2.46498000 |
| C | 1.51616700 | 2.64623200 | 1.96165100 |
| C | 0.69714500 | 3.71462600 | -1.52589700 |
| C | -0.66073600 | 3.35196100 | -1.73641600 |
| C | 0.66180900 | 1.62959300 | -2.46529800 |
| C | 1.51612300 | 2.64599800 | -1.96205900 |

| | | | |
|---|---|---|---|
| C | -0.67545300 | 2.07151700 | -2.33826400 |
| P | 3.48003300 | -0.24976400 | 1.53873200 |
| P | 3.48023200 | -0.24962400 | -1.53859100 |
| H | 1.04981600 | 4.65952800 | 1.11797500 |
| H | -1.54443700 | 3.94241700 | 1.49509000 |
| H | -1.57869000 | 1.53662300 | 2.63502200 |
| H | 1.00477300 | 0.68785700 | 2.88795000 |
| H | 2.60332800 | 2.59643000 | 1.93296700 |
| H | 1.05010600 | 4.65932000 | -1.11834400 |
| H | -1.54431800 | 3.94240400 | -1.49507200 |
| H | 1.00442700 | 0.68765500 | -2.88826700 |
| H | 2.60328300 | 2.59614300 | -1.93355000 |
| H | -1.57890700 | 1.53669800 | -2.63517200 |
| Fe | 5.17099600 | -0.17185000 | 0.00020800 |
| P | 4.48673700 | -2.11148400 | 1.08158200 |
| P | 4.48690500 | -2.11133600 | -1.08149400 |
| C | 6.76673200 | 0.37939500 | -1.16015000 |
| C | 7.19680400 | -0.34699600 | -0.00062100 |
| C | 6.76768800 | 0.37813400 | 1.16004300 |
| C | 6.06627400 | 1.54615600 | 0.71539800 |
| C | 6.06565400 | 1.54693300 | -0.71366500 |
| C | 7.00902700 | 0.00866000 | -2.58468800 |
| C | 7.97680000 | -1.61783200 | -0.00166000 |
| C | 7.01116400 | 0.00594500 | 2.58400200 |
| C | 5.50366400 | 2.61072000 | 1.59564400 |
| C | 5.50232400 | 2.61247100 | -1.59226300 |
| H | 6.14033200 | 0.26077300 | -3.21169400 |
| H | 7.88758100 | 0.53517400 | -2.99703600 |
| H | 7.17763400 | -1.07225700 | -2.68898500 |

| | | | |
|---|---|---|---|
| H | 7.73454900 | -2.22659300 | -0.88457700 |
| H | 9.06384200 | -1.42575900 | -0.00260200 |
| H | 7.73619200 | -2.22703300 | 0.88139800 |
| H | 7.17972500 | -1.07509200 | 2.68710200 |
| H | 7.89013200 | 0.53195800 | 2.99611200 |
| H | 6.14304900 | 0.25755200 | 3.21200400 |
| H | 6.27828700 | 3.34647900 | 1.87497800 |
| H | 4.69372700 | 3.15716800 | 1.09180000 |
| H | 5.08908500 | 2.18605400 | 2.52114900 |
| H | 4.69225100 | 3.15783000 | -1.08745200 |
| H | 6.27653200 | 3.34897400 | -1.87079700 |
| H | 5.08773600 | 2.18888100 | -2.51825600 |
| H | -7.88973000 | -0.53138300 | 2.99640700 |

$[(Cp_2Hf)_2(P5)_2(FeCp^*)_2]^{\cdot-}$

-1 2

| | | | |
|---|---|---|---|
| Fe | 5.15195700 | -0.19571300 | -0.01049600 |
| C | 6.03559400 | 1.49975000 | 0.77558600 |
| C | 6.02336000 | 1.56134900 | -0.65196100 |
| P | 3.46954600 | -0.24659700 | -1.56268800 |
| C | 6.73361000 | 0.42151700 | -1.15310000 |
| P | 4.49523900 | -2.10959900 | -1.14680800 |
| C | 7.17975600 | -0.34896700 | -0.02840700 |
| P | 4.47941600 | -2.17198300 | 1.01351800 |
| C | 6.75191100 | 0.32158500 | 1.16493100 |
| P | 3.45610700 | -0.33322900 | 1.51883300 |
| Hf | 0.24305800 | 1.82607100 | -0.00406800 |
| C | 0.47702100 | 3.77992000 | -1.51154700 |

| | | | |
|---|---|---|---|
| C | -0.79960000 | 3.22268900 | -1.77920500 |
| C | -0.59617000 | 1.95790600 | -2.38542200 |
| C | -0.40154000 | 3.59023900 | 1.61468000 |
| C | 5.46931100 | 2.52081600 | 1.70378800 |
| P | 1.89404000 | -0.24073800 | -0.02700200 |
| C | 5.44094300 | 2.65848300 | -1.47728000 |
| C | 6.96935000 | 0.11454900 | -2.59376700 |
| C | 7.97336800 | -1.60994700 | -0.08786700 |
| C | 7.00846600 | -0.10888900 | 2.57006500 |
| H | 4.63100000 | 3.06063700 | 1.24107900 |
| H | 6.23241000 | 3.26744300 | 1.98624600 |
| H | 5.09260600 | 2.05662000 | 2.62637600 |
| H | 5.02482600 | 2.27286500 | -2.41895900 |
| H | 6.20332300 | 3.41788700 | -1.72524100 |
| H | 4.62753400 | 3.16745200 | -0.94069400 |
| H | 6.09435000 | 0.38686400 | -3.20331000 |
| H | 7.14515500 | -0.95961700 | -2.74593300 |
| H | 7.84117800 | 0.66502600 | -2.98865000 |
| H | 7.73836800 | -2.26249300 | 0.76530100 |
| H | 9.05827500 | -1.40640400 | -0.07748300 |
| H | 7.73904100 | -2.17916000 | -0.99877600 |
| H | 6.13993900 | 0.10150400 | 3.21250700 |
| H | 7.88149200 | 0.41204400 | 3.00084100 |
| H | 7.19390400 | -1.19061600 | 2.62497600 |
| H | 0.66366100 | 4.75901100 | -1.07450700 |
| C | 1.46515300 | 2.84464700 | -1.91434400 |
| H | -1.77287700 | 3.66692200 | -1.57022300 |
| H | -1.40164900 | 1.30160400 | -2.71768000 |
| C | 0.79341500 | 1.72203100 | -2.46627600 |

| | | | |
|---|---|---|---|
| H | 1.29185800 | 0.84260400 | -2.87261800 |
| H | 2.54546700 | 2.95585300 | -1.83694100 |
| C | 1.01753400 | 3.64011900 | 1.50914200 |
| H | 1.60549400 | 4.45861200 | 1.09690600 |
| C | 1.54255000 | 2.44572500 | 2.04920600 |
| H | -1.10605000 | 4.35263200 | 1.28356400 |
| C | -0.74987000 | 2.37062600 | 2.23779100 |
| H | -1.76408900 | 2.03909000 | 2.46209400 |
| C | 0.44770700 | 1.65442700 | 2.48914700 |
| H | 0.52888600 | 0.68353700 | 2.97026200 |
| H | 2.59226200 | 2.16754900 | 2.12241600 |
| Hf | -0.24304800 | -1.82604400 | 0.00400900 |
| P | -1.89407700 | 0.24077300 | 0.02720600 |
| C | -0.47297100 | -3.78088400 | 1.51088300 |
| C | 0.80202700 | -3.22051000 | 1.77945600 |
| C | 0.59497400 | -1.95634700 | 2.38582500 |
| C | -0.79524500 | -1.72405300 | 2.46597500 |
| C | -1.46375900 | -2.84818000 | 1.91327000 |
| C | -1.01408200 | -3.64157400 | -1.50906500 |
| C | 0.40463300 | -3.58706800 | -1.61694800 |
| C | 0.74790300 | -2.36597800 | -2.24005300 |
| C | -0.45233600 | -1.65347700 | -2.48886500 |
| C | -1.54387100 | -2.44845000 | -2.04732800 |
| P | -3.46964300 | 0.24749700 | 1.56283500 |
| P | -3.45614100 | 0.33192400 | -1.51871400 |
| H | -0.65680900 | -4.76038000 | 1.07357700 |
| H | 1.77656900 | -3.66218600 | 1.57092900 |
| H | 1.39858400 | -1.29811200 | 2.71874900 |
| H | -1.29615700 | -0.84592800 | 2.87213700 |

| | | | |
|---|---|---|---|
| H | -2.54373500 | -2.96222000 | 1.83524100 |
| H | -1.59869400 | -4.46223500 | -1.09640300 |
| H | 1.11223400 | -4.34730600 | -1.28745900 |
| H | 1.76074500 | -2.03125500 | -2.46591700 |
| H | -0.53770000 | -0.68251200 | -2.96911000 |
| H | -2.59454500 | -2.17343100 | -2.11849800 |
| Fe | -5.15206800 | 0.19557100 | 0.01061600 |
| P | -4.49545300 | 2.11016900 | 1.14558700 |
| P | -4.47931700 | 2.17114700 | -1.01477500 |
| C | -6.03610400 | -1.50014900 | -0.77460000 |
| C | -6.02349200 | -1.56113200 | 0.65297400 |
| C | -6.73351900 | -0.42101200 | 1.15378800 |
| C | -7.17985700 | 0.34903200 | 0.02886500 |
| C | -6.75235400 | -0.32206800 | -1.16427900 |
| C | -5.47033300 | -2.52169800 | -1.70257400 |
| C | -5.44089100 | -2.65790400 | 1.47864500 |
| C | -6.96890300 | -0.11340500 | 2.59437500 |
| C | -7.97334200 | 1.61010900 | 0.08794000 |
| C | -7.00921400 | 0.10780100 | -2.56954400 |
| H | -4.63268600 | -3.06225300 | -1.23953300 |
| H | -6.23396500 | -3.26768100 | -1.98528300 |
| H | -5.09289800 | -2.05784300 | -2.62504000 |
| H | -5.02415700 | -2.27181300 | 2.41985800 |
| H | -6.20328500 | -3.41701900 | 1.72744700 |
| H | -4.62785500 | -3.16732500 | 0.94192300 |
| H | -6.09378800 | -0.38554200 | 3.20383500 |
| H | -7.14457900 | 0.96084200 | 2.74612000 |
| H | -7.84069100 | -0.66363500 | 2.98968900 |
| H | -7.73834800 | 2.26232300 | -0.76548500 |

| | | | |
|---|---|---|---|
| H | -9.05826900 | 1.40666200 | 0.07771300 |
| H | -7.73888700 | 2.17963000 | 0.99862300 |
| H | -6.14092000 | -0.10308100 | -3.21213700 |
| H | -7.88247000 | -0.41315800 | -2.99982600 |
| H | -7.19444500 | 1.18954000 | -2.62491400 |

## IX. References


1. O. J. Scherer and T. Brück, *Angew. Chem. Int. Ed.*, 1987, **26**, 59-59.
2. S. Stoll and A. Schweiger, *J. Magn. Reson.*, 2006, **178**, 42-55.
3. G. Sheldrick, *Acta Crystallogr. A*, 2015, **71**, 3-8.
4. G. Sheldrick, *Acta Crystallogr. C*, 2015, **71**, 3-8.
5. O. V. Dolomanov, L. J. Bourhis, R. J. Gildea, J. A. K. Howard and H. Puschmann, *J. Appl. Crystallogr.*, 2009, **42**, 339-341.
6. B. Ravel and M. Newville, *J Synchrotron Radiat*, 2005, **12**, 537-541.
7. S. Grimme, J. Antony, S. Ehrlich and H. Krieg, *J. Chem. Phys.*, 2010, **132**.
8. F. Weigend and R. Ahlrichs, *PCCP*, 2005, **7**, 3297-3305.
9. C. Adamo and V. Barone, *J. Chem. Phys.*, 1999, **110**, 6158-6170.
10. M. J. Frisch, G. W. Trucks, H. Schlegel, G. E. Scuseria, M. A. Robb, J. R. Cheeseman, G. Scalmani, V. Barone, G. A. Petersson and H. Nakatsuji.
11. E. D. Glendening, C. R. Landis and F. Weinhold, *J. Comput. Chem.*, 2019, **40**, 2234-2241.
12. T. Lu, *J. Chem. Phys.*, 2024, **161**.
13. T. Lu and F. Chen, *J. Comput. Chem.*, 2012, **33**, 580-592.
